# A digital health game to prevent opioid misuse and promote mental health in adolescents in school-based health settings: Protocol for the *PlaySmart* game randomized controlled trial

**DOI:** 10.1371/journal.pone.0291298

**Published:** 2023-09-08

**Authors:** Tyra M. Pendergrass Boomer, Lily A. Hoerner, Claudia-Santi F. Fernandes, Amber Maslar, Sherry Aiudi, Tassos C. Kyriakides, Lynn E. Fiellin

**Affiliations:** 1 play2PREVENT Lab at the Yale Center for Health & Learning Games, Department of Internal Medicine, Yale School of Medicine, New Haven, CT, United States of America; 2 Yale Child Study Center, New Haven, CT, United States of America; 3 Department of Biomedical Informatics and Data Science, Yale School of Medicine, New Haven, CT, United States of America; 4 Memorial Sloan Kettering Cancer Center, New York, NY, United States of America; 5 Yale Center for Analytical Sciences, Yale School of Public Health, New Haven, CT, United States of America; 6 Yale School of Public Health, New Haven, CT, United States of America; Public Library of Science, UNITED KINGDOM

## Abstract

Adolescents who engage in non-opioid substance misuse and/or experience mental health symptoms are at greater risk of misusing opioids and/or developing opioid use disorder. Adolescence is a critical developmental period to both prevent the initiation of opioid misuse and target mental health. To date, there are no digital health games targeting both conditions. We describe the protocol for a randomized controlled trial designed to assess the efficacy of an original digital health game, *PlaySmart*. Five hundred and thirty-two adolescents aged 16–19 years old, who are at greater risk for initiating opioid misuse are recruited from 10 Connecticut school-based health sites. Participants are randomized to *PlaySmart* or a set of time/attention control videogames. Randomization was stratified by sex at birth and school grade. Participants play their assigned game or games for up to six weeks (300 minutes) and complete assessment questions over a 12-month period (baseline, post-gameplay, 3, 6, and 12 months). The primary outcome is perception of risk of harm of opioid misuse at 3 months. Secondary outcome measures specific to opioid misuse include intentions, self-efficacy, attitudes, knowledge, and perceived norms. Mental health outcomes include measures of depression (Patient Health Questionnaire-8), anxiety (Generalized Anxiety Disorder-7), help-seeking behaviors, stigma, measures of self-regulation, self-efficacy to seek professional help for mental health, and knowledge around coping skills. *PlaySmart* has the potential to significantly reduce the risk of initiation of opioid misuse, improve mental health outcomes, and given its high levels of engagement and accessibility, holds the promise for extensive reach, scale, and impact for adolescents.

**Trial registration**: ClinicalTrials.gov: NCT04941950. Registered on 23 June 2021.

## Background

Opioids are a potentially addictive class of drugs that are legally available by prescription as pain relievers and illegally as drugs such as heroin [1]. Since the severity of the opioid epidemic has risen to a national public health emergency [2], prescribing opioids has become a cause for concern [3]. Reducing rates of opioid prescribing to adolescents helps to minimize the potential misuse of prescription opioids and associated health consequences, as adolescents who report opioid misuse often report medical use of opioids prior to misuse [4]. Forty percent of prescription opioid misuse and 68% of heroin misuse begins during adolescence [5], and the risk of developing opioid use disorder (OUD) and other substance use disorders (SUDs) is heightened during adolescence [6]. According to the 2019 Youth Risk Behavior Survey (YRBS) that included a sample of 13,872 participants from 136 different US schools, 14% of high school students reported lifetime misuse of opioids and half of those reported currently misusing opioids [7, 8]. Misuse of prescription opioids among adolescents is also associated with other adverse health outcomes and risk behaviors, including the use of alcohol and other drugs, youth violence, increased risk for HIV and other sexually transmitted diseases, and an increased risk of overdose [9]. Drug overdose is now the leading cause of death for those under age 50, and 75% of these 92,000 deaths were opioid-related in 2020 alone [10].

Studies have also demonstrated that opioid misuse or non-prescription use of opioids is highly correlated with mental health issues [11, 12]. Mental health and its relationship to opioid misuse must be examined and addressed through preventive interventions, as mental health issues in youth can precede a multitude of other problems, including but not limited to delinquency [13], suicide [14], mood and anxiety disorders [12], opioid misuse and OUD [11, 12], and other SUDs [13, 15]. Recently, the COVID-19 pandemic has exacerbated the already troubling youth mental health crisis, leading to a call for action from the Office of the U.S. Surgeon General [14, 16]. Even before the pandemic in 2019, one in three high school students had reported persistent feelings of sadness and hopelessness, representing a 40% increase from 2009 [15]. Reflecting the severity of the increase in mental health issues, suicide is now the second-leading cause of death for those ages 15–24, with 36% seriously considering attempting [17]. Adolescents are at a critical period in their development where education and interventions to address mental health issues are imperative and can potentially assist in preventing or reducing the risk for opioid misuse. Preventive interventions that target distinct issues that are often interrelated, such as mental health and opioid misuse, allow for a greater efficacy of addressing these issues and a wider overall impact.

In 2018, the National Institutes of Health launched the Helping to End Addiction Long-term (HEAL) Initiative to combat the opioid crisis, outlining 26 research priorities that target unmet needs for improving treatment for OUD and preventing opioid misuse in higher risk populations [6, 18]. Ten projects, including the study described here (1UG3DA050251-01/4UH3DA050251-03; PI: Fiellin), around the country are a part of the HEAL Prevention Cooperative (HPC) and are funded by the HEAL Initiative, and all of these target different populations that are at higher risk of opioid misuse in a variety of settings [6, 18–21].

Videogames as interventions, or digital health games, have the advantage of meeting adolescents “where they are,” physically, educationally, and through means they are already familiar with–technology. Over 90% of adolescents play video games [22]. Serious games–games that have a primary purpose other than solely entertainment–are an ideal prevention platform, as they give adolescents the opportunity to practice healthy behaviors before transferring these skills to the real world [23, 24]. Unlike other interventions, serious games can be delivered with consistent fidelity; they also have the advantage of requiring minimal additional personnel and support while being easily delivered and distributed. The play2PREVENT^®^ Lab has a wealth of experience in developing, evaluating, and disseminating easily accessible videogame interventions that target a range of health behaviors in adolescents [25–37].

As a part of the HEAL Initiative, our research team developed and is evaluating a digital health game, *PlaySmart*, that aims to prevent the initiation of opioid misuse and promote mental health by increasing the perception of risk of harm from opioid misuse and increasing adaptive mental health strategies, respectively [6, 18]. The *PlaySmart* formative work incorporated input from adolescents to develop the intervention. There is strong evidence that opioid misuse and non-medical prescription opioid use are highly correlated with mental health issues [11]. This is further supported by the fact that the themes of mental health and seeking support emerged from our focus group discussions when gathering input on how to prevent the initiation of opioid misuse in adolescents. The subsequent pilot study used feedback from adolescents to establish the acceptability of the intervention with the target population.

The current randomized controlled trial (RCT) is evaluating the impact of the *PlaySmart* game, as compared with a control condition, on a range of outcomes related to opioid misuse and mental health in older adolescents at school-based health sites. Given its evidence-based development, mode of delivery, engagement, and accessibility, *PlaySmart* has the potential to both prevent opioid misuse and promote positive mental health behaviors and have considerable reach and impact on its target population.

## Methods

### Study overview

This study is a RCT designed to assess the efficacy of an original digital health game, *PlaySmart*, that targets opioid misuse and mental health in older adolescents. Five hundred and thirty-two adolescents, 16–19 years old, who are at higher risk for opioid misuse are recruited from 10 Connecticut high schools. These high schools partner with us through school-based health programs, working with School-Based Health Centers (SBHCs), school-based health staff, or other integral school personnel. Participants are randomized 1:1 to either the intervention group where they play *PlaySmart* or a set of time and attention control videogames that include games such as *That Dragon Cancer*, *Can You Escape*, and *The Sims*. These games have no relevant content to our target outcomes. For sites that have SHBCs, their staff and the research study team work together for recruitment and screening efforts. Recruitment efforts include the use of the lunch wave process (tabling and providing opt-outs to interested students), posters with QR codes, morning school announcements, and videos that detail the project.

### Study timeline

**Fig 1:**
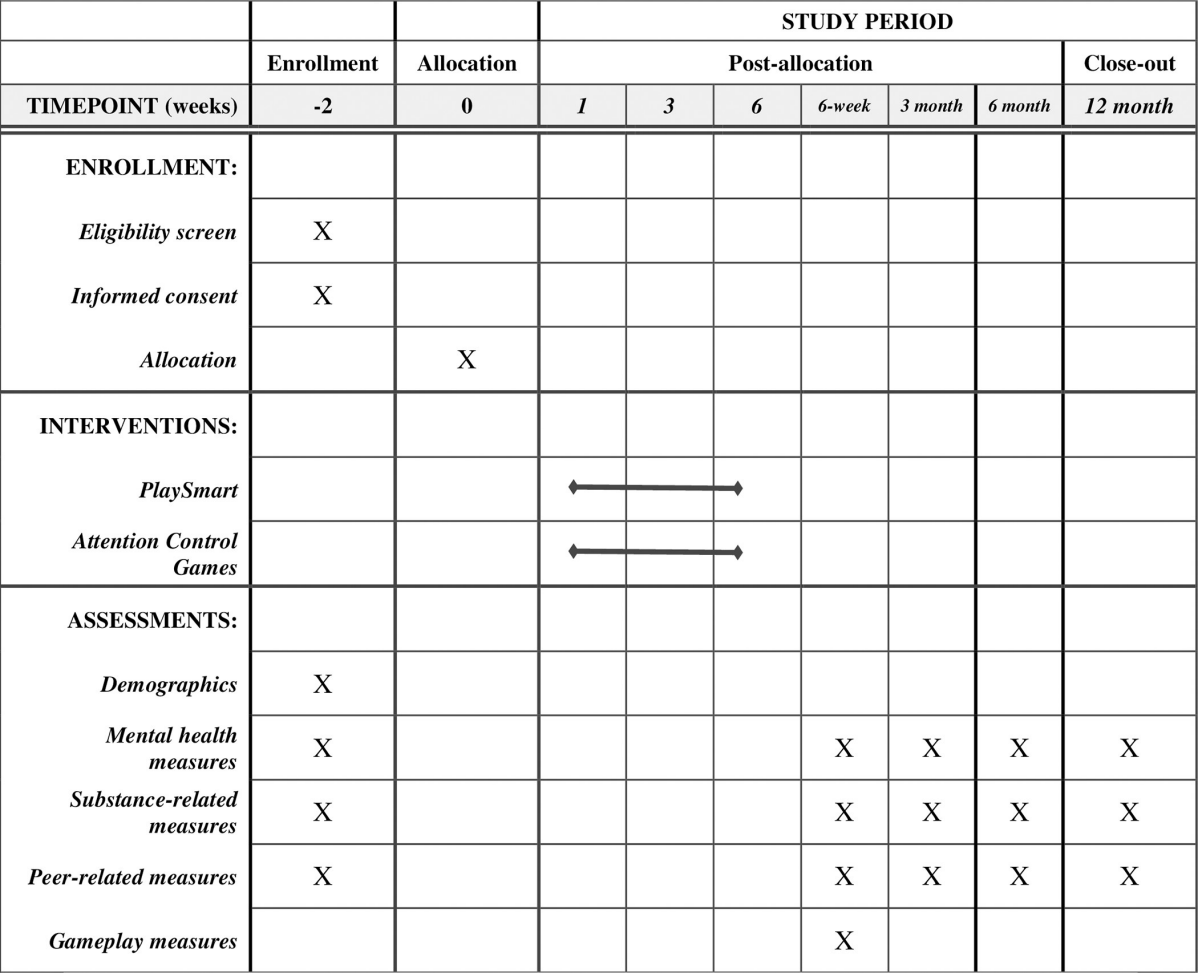
The study timeline, an adaptation of the SPIRIT figure.

Recruitment and enrollment for this RCT commenced in the Fall of 2021 on October 21^st^, 2021 and will continue through the Fall of 2023 ending on December 14^th^, 2023, and the 12-month follow-up will continue through the Fall of 2024. During the summers, the research team focuses its efforts on retention for the follow-up assessments, as no enrollment or intervention delivery takes place when school is not in session. The study timeline, an adaptation of the SPIRIT figure, can be found in Fig 1.

#### Recruitment, screening, eligibility, and informed consent/assent

Interested participants are given a brochure about the study and an opt-out form. The opt-out form was created for the study to inform parents/guardians ahead of the eligibility screening process for the project, given that the screener includes questions about anxiety, depression, and substance misuse. Research study staff distribute these materials to interested potential participants during on-site recruitment sessions or partnered school-based health program staff distribute the form to students directly or electronically to parents/guardians of 16- and 17-year-olds who are enrolled in their health program. These forms are distributed ahead of all screenings, and the research team follows up with the potential participant via text to ensure that the form and brochure have been given to their parent/guardian. If a parent/guardian does not return the signed opt-out form indicating that their teen to be screened for the study, the school-based health program staff or research study team proceeds with screening the participant for study eligibility. If a student or parent/guardian does return the signed opt-out form, the research team documents this, and the student is never screened for eligibility. Students who are 18 or 19 years old receive a version of the opt-out form that is tailored to them and that does not require that it be shown to a parent/guardian.

The screening tool used to assess eligibility of potential participants consists of 14 questions that assess substance use and levels of anxiety and depression. Screeners are completed by potential participants independently under the supervision of school health staff or the research team. The composite screening tool was developed for this project and uses screening questions from CRAFFT N+ [38], Generalized Anxiety Disorder 2-item scale (GAD-2) [39], and Patient Health Questionnaire 2-item scale (PHQ-2) [39] which are commonly used in school-based health settings. Eligibility criteria for study participation are: 1) preferable enrollment in a SBHC; 2) aged 16–19 (grades 9–12); 3) report of no prior opioid misuse; 4) self-report past 30-day use of cigarettes, e-cigarettes, Juul, alcohol, marijuana (including synthetics), amphetamine, cocaine, benzodiazepines, ecstasy, bath salts, or any other misuse of non-opioid prescription drugs or use of non-opioid illicit drugs and/or have a score of ≥ 1 on the PHQ-2 [39] OR a score of ≥ 1 on the GAD-2 [39]; 5) willingness to sit for 60 minutes/session to play the game; and 6) ability to provide assent and parental/guardian consent (if under age 18).

If a potential participant is found to be eligible, they are given an enrollment packet that contains a study timeline and consent form that they must sign (if they are 18 years or older), or their parent/guardian must sign if they are under the age of 18. The research team follows up with the potential participant and parent/guardian (when appropriate) as a reminder to return the completed forms. Parent/guardian consent forms in Spanish, Portuguese, and French are provided when appropriate for the primary language spoken at home. A full ethical and human subjects research review was conducted and approved by the Pediatric Protocol Review Committee and the Institutional Review Board at the Yale School of Medicine (Protocol # 2000030553).

For our partner schools that have school-based health programs (including SBHCs), we work with clinical and non-clinical staff to screen and enroll students. Any mental health or substance use concerns that arise from participants taking the screener are addressed by clinicians in the school-based health programs. If any potential participant is found to be ineligible; they receive a packet that contains informational flyers developed by National Institute on Drug Abuse (NIDA), Substance Abuse and Mental Health Services Administration (SAMHSA), and our lab that provides information on drugs and the teen brain, mental health, school-based health programs, and school counselors. For schools without school-based health programs, we work with other integral school staff (e.g., health teachers, principals, and security guards) to enroll students. All students (those enrolled and not enrolled in the study) are provided with a packet that contains information mental health, substance use, and resources available to them in their school either after screening (if ineligible) or after baseline (if eligible). During the first baseline meeting (see below) prior to study enrollment and data collection, the components of the research project are explained again to participants and adolescent assent is obtained for those participants under 18 who turned in their parent/guardian consent form.

#### Baseline assessment meetings

Following obtaining assent and consent, baseline assessment meetings are conducted in-person at the partner school sites and divided into two days to facilitate on the ground workflow. The research study team enters the participant’s contact information into the *REDCap P11* database to set them up with a *REDCapPRO* account. The database sends a personalized link for the participant to set up an electronic account on *REDCapPRO* that is then used to complete their assessment questions at all timepoints associated with the study. *REDCap (P11)* is a highly secure version of *REDCap*, the 21 CFR Part 11 Validated *REDCap* Service that meets the required Food and Drug Administration (FDA) compliance guidelines for this study, and *REDCapPRO* is an extension of *REDCap(P11)* that allows participants to access their assessments so that data is populated into *REDCap(P11)* by the participant [40, 41].

During the second baseline assessment meeting, held one to two days later, participants complete baseline assessment questions electronically through their *REDCapPRO* account, which is the preferred method, or on paper and the research study team enters them into the study’s *REDCap(P11)* database [40, 41]. The study initially began with all participants completing baseline on paper as the database was being developed to fit the study’s workflow. During the 2022–2023 school year, the team made the transition to collecting all data electronically (except in extreme cases where technology prevented this, then paper assessments were still used).

Participants receive a $55 gift card upon completion of baseline assessments. Additionally, each participant who completes the baseline assessment receives a packet of informational flyers developed by NIDA, SAMHSA, and our lab that provides information on drugs and the teen brain, mental health, and school-based health programs and school counselors (similar to participants deemed to be ineligible for the study).

#### Randomization

After completion of the baseline assessments and prior to participants’ first gameplay session, participants are randomized by research study staff. A single randomization scheme was generated and written in *REDCap(P11)*. Randomization is stratified by sex at birth (male and female) and grade (dichotomized to 9–10 or 11–12). Eligible participants are assigned to play either *PlaySmart* or to a menu of control games on an iPad.

#### PlaySmart experimental condition

Participants that are randomized to the intervention condition are assigned to play the digital health game *PlaySmart*. Components of *PlaySmart* are based on evidence-based behavior change theories; these are embedded throughout the six storylines and five minigames. Each storyline targets one or more unique themes that emerged during the development work with the target audience of adolescents and other stakeholders: exposure to opioids (both in a medical setting and in an informal, recreational setting), accessing mental health treatment, peer-to-peer support, boundaries with peers, reasons to misuse, and support systems.

#### Attention/Time control condition

Participants that are randomized to the control condition have their choice of playing nine videogames such as *That Dragon Cancer*, *Papers Please*, and *The Sims*. These control games contain no relevant content related to the study goals.

#### Gameplay sessions

Participants play on assigned iPads for gameplay sessions one to two times a week for 40 minutes to an hour over 3–6 weeks (depending on individual school site schedule). Gameplay sessions occur mostly after school, but at certain school sites, the school allows the study team to conduct gameplay sessions during participant study halls or free periods. The research study staff bring assigned iPads, headphones, and snacks to each gameplay session. The target gameplay time for the gameplay intervention is 300 minutes, regardless on the overall timeframe for gameplay sessions.

#### Follow-up time periods

Outcome measures are being collected at baseline, post-gameplay (immediately following completion of gameplay), 3, 6 and, 12 months. The primary outcome is the perception of risk of harm of opioid misuse at the 3-month time-point. We also collect data on secondary outcomes and gameplay experience. Data on gameplay experience are only collected for experimental participants at the post-gameplay timepoint through a series of intervention-specific gameplay questions as well as qualitative interviews with those participants. Each assessment time-point has a specified window within which collection of the assessment data are considered “on time” as indicated: 6-week and 3-month assessments (+/-2 weeks); 6-month and 12-month assessments (+/- 1 month). Participants receive gift cards for completing assessments at each study visit: baseline ($55), post gameplay ($35), 3-month ($45), 6-month ($45), 12-month ($45). For follow-up timepoints during the school year, sessions are held in person at partner high school sites and are coupled with a pizza party for participants. For assessments that fall outside of the school year, research study staff meet with participants online via Zoom to be available for any questions that may arise as they answer their questions electronically.

#### Participant retention

A total of 532 participants, aged 16–19 from 10 high schools including those that have school-based health programs are currently being recruited for this RCT to test the efficacy of *PlaySmart*. The research study team is highly engaged with participants to ensure text reminders are sent before and the day of all gameplay sessions and assessment completion days.

#### Data collection and management

Study data is collected and managed using *REDCap* electronic data capture tools hosted at Yale University [40, 41]. *REDCap* (Research Electronic Data Capture) is a secure, web-based software platform designed to support data capture for research studies, providing 1) an intuitive interface for validated data capture; 2) audit trails for tracking data manipulation and export procedures; 3) automated export procedures for seamless data downloads to common statistical packages; and 4) procedures for data integration and interoperability with external sources. *REDCap(P11)* requires enrolled participants to set up accounts via *REDCapPRO* to answer their assessment questions associated with the study; this ensures that the intended participant is the only one who can answer the assessment questions. When this study began, we utilized the paper version of the study assessments to collect data, as the *REDCap(P11)* database was being completed and tailored to our study. After collecting participants’ assessment data on paper, the study team would enter the data into the backend of *REDCap(P11)*. During the summer of 2022, the project transitioned to fully electronic versions of the assessments that can be emailed out to participants through the *REDCapPRO* extension of the *REDCap(P11)* system. This allows study participants to have their answers directly recorded in the database without the study team having to manually enter the data points.

We have established a Data and Safety Monitoring Board (DSMB) comprised of three experts in clinical trials in adolescents (one with expertise in substance use prevention), an expert in statistical analysis of clinical trials, and an expert in intervention design and implementation of RCTs related to opioids and other substances. The DSMB meets with the research team twice yearly to review study progress and review any specific challenges or adverse events.

#### Data measures

Data from assessment measures help evaluate changes in the primary and secondary study outcomes. These measures are a compilation of both assessments that are used across all 10 HEAL project sites (“common measures” harmonized across all sites) and ones that are specific to our study site. A complete description of all assessment measures by timepoint can be found in **Table 1**.

**Table 1:** Timeline of assessment and corresponding data measures.

Assessment	Baseline	Week 6	Month 3	Month 6	Month 12
**Demographics**	**X**				
**Involvement with Substances**	**X**	**X**	**X**	**X**	**X**
**Self-efficacy to Refuse and Not Misuse**	**X**	**X**	**X**	**X**	**X**
**Mental Health (Depression and Anxiety)**	**X**	**X**	**X**	**X**	**X**
**Pain**	**X**	**X**	**X**	**X**	**X**
**Social Exposure**	**X**	**X**	**X**	**X**	**X**
**Perception of Risk of Harm**	**X**	**X**	**X**	**X**	**X**
**Intentions to Misuse**	**X**	**X**	**X**	**X**	**X**
**Perceived Norms**	**X**	**X**	**X**	**X**	**X**
**Decision Making Skills**	**X**	**X**	**X**	**X**	**X**
**Emotion Regulation**	**X**	**X**	**X**	**X**	**X**
**Knowledge about Opioid Misuse/Risks**	**X**	**X**	**X**	**X**	**X**
**Help Seeking Behaviors**	**X**	**X**	**X**	**X**	**X**
**Self-Stigma of Seeking Help Scale**	**X**	**X**	**X**	**X**	**X**
**Beliefs About Psychological Services**	**X**	**X**	**X**	**X**	**X**
**Gameplay Questions**		**X**			
**In-game Data**		**X**			

*Demographics*. Demographic characteristics such as age, grade level, sex at birth, gender identity, sexual orientation, race, ethnicity, education level, and socio-economic status are collected through 12 questions.

*Involvement with substances*. Involvement with four different substances (e.g., illegal and legal opioids, alcohol, and marijuana) is assessed through four stem question asking about lifetime use inside and outside of medical settings for each substance. Measures of involvement with all substances are harmonized and the same instrument is used across all 10 HEAL sites [20, 21, 42, 43]. If participants answer yes to lifetime use for any of the substances, they are asked 11 questions about frequency and consequences.

*Emotional regulation*. Self-regulation in a specific moment (i.e. trait) is assessed only once at baseline with the Difficulties in Emotional Regulation Scale (DERS), containing 10 questions [44]. Self-regulation across time (i.e. state) is assessed at all timepoints and with use of The State Difficulties in Emotion Regulation Scale (S-DERS), a 10-item inventory [45]. Answer choices on the DERS range from *not at all* to *completely*, while answer choices on the S-DERS range from *not at all* to *almost always*. Measures of emotional regulation are not harmonized across all HEAL sites.

*Self-efficacy*. Self-efficacy to both do not misuse and to refuse prescription opioids and heroin are assessed through a total of 8 questions on two separate forms. Participants are given the stem, “Imagine that you have access to prescription opioids/heroin…” with branched questions asking about confidence to not misuse and refuse prescription opioids and heroin in the next 30 days. They are given 5 answer choices ranging from *not at all* to *extremely*. These measures were developed from the Drug Use Resistance Self-efficacy (DURSE) Scale [46]. The measure looking at the self-efficacy to misuse is harmonized and used across all HEAL sites [20]. Self-efficacy to refuse is a study-specific measure not harmonized across HEAL sites.

*Mental health*. Both anxiety and depression are measured at all timepoints through the use of the Patient-Health Questionnaire 8-item (PHQ-8) for depression [47] and the Generalized Anxiety Disorder 7-item inventory (GAD-7) for anxiety [48]. For each of measures, participants are given the stem question: “Over the last two weeks how often have you been bothered by any of the following problems…” with 8 question descriptors for depression (PHQ-8) and 7 question descriptors for anxiety (GAD-7). Measures of mental health are harmonized and used across all HEAL sites [20].

*Pain*. Experience with pain is assessed with the PROMIS Pain Interference Scale (PROMIS-PI) 2 item scale; answer choices range from *Never* to *Almost Always* [49, 50]. The PROMIS-PI measure is harmonized and used across all HEAL sites [20].

*Social exposure and peer norms*. Social exposure to substance misuse is assessed with the stem question: “What is the most often that someone who lives with you…” with 3 question descriptors, asking about three different substances: alcohol, marijuana, and heroin. Answer choices range from *Never* to *4–7 days a week* [51]. Peer norms are assessed similarly through two questions related to the prevalence of opioids misuse in their school, with answer choices ranging from *hardly any or none* to *all or most* [52]. Peer norms is a study-specific measure not harmonized across all HEAL sites.

*Perception of risk of harm of opioid misuse scale*. Perception of risk of harm of opioid misuse is the study’s primary outcome. There is a well-established inverse relationship between perception of risk of harm from drug use and actual drug use [53]. This relationship in which low perceived harmfulness is strongly associated with a high risk of misuse has been demonstrated with non-medical use of prescription drugs [54], prescription opioids [55–57], and heroin [58]. Individuals with low perceived harmfulness were almost 10 times more likely to use prescription opioids nonmedically, as compared to those with high perceived harmfulness (95% CI = 2.1–44.0) [55]. Similarly, results indicate a significant association between reporting great risk of trying heroin and lower likelihood of lifetime heroin use (OR = 0.38, 95% CI: 0.33, 0.44, p < 0.001) [58]. Given there is evidence that youth generally have a low perception of risk of harm around opioid misuse, we focus on perceived risk of harm from opioid misuse as a proximal target underlying the videogame intervention’s impact on the more distal outcome of initiation of opioid misuse.

Perception of risk of harm score (PRHS) is assessed with the stem question: “How much do you think people risk harming themselves (physically or in other ways), if they [branched question]?” with 8 different branched questions related to illegal opioids (heroin) and legally available opioids (e.g., Percocet, OxyContin, etc.). Examples of branched questions include “try heroin once or twice?” and “try any narcotic other than heroin (non-heroin narcotics such as prescription opioids such as Codeine, Vicodin, Oxycontin, Percocet, etc.) once or twice?” Answer choices are *no risk*, *slight risk*, *moderate risk*, and *great risk*, providing a score range of 1–4 [21, 53]. Perception of risk of harm is a study-specific measure not harmonized across HEAL sites.

*Intentions to misuse opioids*. Intentions to misuse opioids is assessed with the stem question: “How likely is it that you will [question descriptor] over the next 12 months?” with four different question descriptors. Examples of question descriptors include “use prescription opioids, even once or twice” and “use heroin, even once or twice.” Answer choices are *definitely not*, *probably not*, *probably will*, and *definitely will* [59]. Intentions to misuse opioids is a study-specific measure not harmonized across HEAL sites.

*Attitudes toward misuse and its risks*. Attitudes toward misuse and its risk are assessed by having participants indicate how much they agree or disagree with 15 different branched prompts with the following stem statement: “If I used/took a prescription opioid, or painkiller, or heroin, I would [branched prompt].” Examples of branched prompts included: feel good, get away from my problems, and feel less anxious. Five answer choices are presented that range from *strongly disagree* to *strongly agree* [60]. Attitudes towards misuse and its risks is a study-specific measure not harmonized across HEAL sites.

*Decision-making skills*. Decision-making skills are assessed by presenting participants with 9 statements such as: “I think about difficult situations that I might find myself in and make plans to deal with them” and “When there is a problem, I try to figure out what caused it.” For these statements, their answer choices are *never*, *seldom*, *about half the time*, *often*, and *always* [61]. Decision making skills is a study-specific measure not harmonized across HEAL sites.

*Knowledge about opioid misuse and its risks*. Knowledge is assessed with 30 statements such as “Prescription opioids can be just as deadly as heroin when they are misused”. Participants are given answer choices of *true*, *false*, or *not sure*. This instrument was adapted from the AIDS Risk Behavior Knowledge scale and modified to fit the outcomes around opioids and opioid misuse targeted in our study [62]. Knowledge about opioid misuse and its’ risks is a study-specific measure not harmonized across HEAL sites.

*Help-seeking*. Participants are asked both about their past help-seeking behaviors and general help-seeking. Past Help-Seeking is assessed by presenting the participant with a list of 13 different people that they might seek help or advice from; they check any of those people/resource that they have gone to for advice or help in the past six months. If they sought help from that person/resource, they are then asked to quantify the number of times in the past six months. Examples of people and resources listed include: partner, friend, school psychologist, phone/texting help line, internet [63, 64]. Participants are also given the General Help-Seeking Questionnaire to assess their tendencies to seek help in times of emotional distress [65]. This instrument contains 20 items related to how likely it is that they would seek help if (a) experiencing a personal or emotional problem and (b) if they were experiencing suicidal thoughts. The answer choices are given on a 7-point Likert scale with answers ranging from *extremely unlikely* to *extremely likely*. Both help-seeking measures are study-specific and not harmonized across HEAL sites.

*Self-stigma of help-seeking scale*. Stigma associated with help-seeking for mental health or substance misuse is assessed with the 10-item Self-Stigma of Seeking Help (SSOSH) [66]. Five answer choices are given ranging from *strongly disagree* to *strongly agree*. SSOSH is a study-specific measure not harmonized across HEAL sites.

*Beliefs about psychological services*. The Beliefs About Psychological Services (BAPS) scale is used to assess self-beliefs about seeking help and professional psychological services [67]. The answer choices provided are *true* or *false*. The BAPS scale is a study-specific measure not harmonized across HEAL sites.

*Gameplay experience*, *usability*, *and engagement*. For participants randomized to the experimental condition, gameplay experience, usability, and engagement is assessed with 15 questions such as: “This game helped me learn important things,” and “I felt frustrated with playing *PlaySmart*.” Answer choices range from *strongly disagree* to *strongly agree* [36]. Gameplay measures are study-specific and not harmonized across HEAL sites.

*In-game data*. In-game data is collected through the backend system of the game and available through the server that hosts the game. In-game data reports allow us to analyze the different decisions and choice paths that the player selected in the game.

#### Sample size

The study is powered to detect a 15% absolute difference between control group and intervention group in terms of perceived risk of harm of misuse. The assumption is that at baseline, 32% of participants would report great perception of risk of harm, so at 3 months, it is assumed that the difference between the two study arms would be 15%: the difference in the proportion of participants achieving the primary outcome (reporting great risk of harm from misuse of prescription opioids and heroin/fentanyl) [50% (*PlaySmart)* vs. 35% (control)] with a power of 90% (p-value ≤ 0.05) (Power Analysis Statistical Software (To account for a 16% loss at 3 months based on our *PlayForward* study [28], the sample size is inflated to 532 participants (266 in each arm).PASS); 2008) [68]. A conservative estimate from the literature on drug use prevention interventions and using data from a study examining the impact of an intervention on perceptions of harm of drug use, indicates that a sample size of 454 participants will be required to detect a 15% absolute difference in the proportion of participants achieving the primary outcome. To account for a 16% loss at 3 months based on our *PlayForward* study [28], the sample size is inflated to 532 participants (266 in each arm).

#### Planned analyses

Descriptive statistics of baseline characteristics (mean, median, IQR) and graphs will be used to assess randomization adequacy. Baseline characteristics that are determined not to be equally distributed among the two groups will be considered for covariate adjustment to determine their impact on intervention comparisons. The primary hypothesis is that, at three months, there will be a higher proportion of *PlaySmart* vs. control participants who report greater perception of risk of harm of misuse of prescription opioids AND heroin/fentanyl. The primary comparison will be the main effects of *PlaySmart* vs. control games in terms of the Perception of Risk of Harm Score (PRHS) that was constructed from a set of eight questions (range of score 1–4). The proportion of participants in the two study arms who achieve the primary outcome (PRHS = 32) at three months will be compared. The probability of achieving the primary outcome will be modeled using logistic regression. An unadjusted model (intervention variable only; stratified and non-stratified) and adjusted models (intervention plus other predictor variables) will be used in the analytical approach. In addition to the 3-month comparison, chi-square tests will be used to assess primary outcome proportion differences between the two study arms at 6 weeks, 6 months and 12 months. Additionally, the mean PRHS (continuous) will be compared at the various study time points (6 weeks/3 months/6 months/12 months) using t-tests. Mixed models will be used for longitudinal analysis of the PRHS to assess differences between the two study groups over time. Further analysis will assess differences in the direction of change (positive/negative delta) for each of the eight questions.

Differences in (a) intentions to misuse opioids and (b) self-efficacy for refusing opioids will be compared between the two groups. Means (standard errors) of intention and self-efficacy scores, by group, will be plotted at the various study time points (6 weeks, 3, 6 and 12 months). Change from baseline at each timepoint will also be compared between the two groups. Mixed Models (MM) will be used for longitudinal analysis to assess differences between the two groups over time in terms of (a) intention and (b) self-efficacy scores.

While the goal of this work is to prevent the initiation of opioid misuse in youth, it is not likely that a substantial proportion of even these adolescents at higher risk will initiate opioid misuse during the 12-month follow-up period. We will compare the proportions of participants initiating opioid misuse at all follow-up timepoints (with a focus on 12 months) using chi-square analyses. Given the observed rate of initiating opioid misuse in high school populations is estimated to be between 7–14% [15, 69], and we are focusing on higher risk adolescents who report current substance use and symptoms of depression or anxiety, we estimate a three-fold increased likelihood of initiating opioid misuse resulting in 42% of the population initiating opioid misuse. Given the relationship between perception of risk of harm and initiation of misuse and a 15% reduction in initiation of opioid misuse based on exposure to the videogame intervention, we expect an absolute decrease in initiation of 6% (36% in the *PlaySmart* arm vs. 42% in the control arm) at 12 months. The effective sample size of 532 will provide 29% power (two-sided alpha-level 0.05) to detect this difference. The probability of achieving this outcome will also be modeled using logistic regression analysis. Stratified (using randomization stratification variables; gender and grade) analysis and non-stratified analyses and adjusted models (e.g., intervention plus other predictor variables, mediator, and moderator variables) will be used in further logistic regression analyses.

Data will be collected on potential mediators and moderators. Since these variables might change over time during participation in the study, they will also be considered as time varying covariates.

Data from secondary outcomes will be presented descriptively at study time points at which they are collected. Numbers and proportions of participants will be presented for each of the components used to assess secondary outcomes. In addition, for standardized tools that have validated and published scoring algorithms, those algorithms will be used to compare the two study arms, both at various time points as well as over time. Additional analyses will be carried out for other subsets of questions in such tools using appropriate methodology to establish groups of questions to analyze together (e.g., Cronbach’s alpha analysis). As appropriate, repeated measures analysis will also be used to compare the two study arms in terms of secondary outcomes. Below the study’s secondary outcomes are classified as mediators or moderators.

*Mediators*. We predict that 1) knowledge, 2) perceived norms, and 3) attitudes will mediate the effects of the intervention on perception of risk of harm from misuse of opioids at 3 months. We will test the relationships among the outcomes and presumed mediators as proposed by MacKinnon et al. [70]. To test the significance of the mediation effect, we will calculate Sobel’s test for each proposed mediator. Sobel’s test determines the significance of the mediation effect by testing the null hypothesis that the indirect effect coefficient is zero (i.e., whether the indirect effect of the independent variable on the dependent variables is significantly different from zero) [71].

*Moderators*. Variables that predict the outcome variable of perceived risk of harm differently between the intervention and control groups will be considered to be moderators using the model outlined by Kraemer et al [72]. To be considered a moderator, the variable must be present prior to randomization and must not be related to the independent variable (*PlaySmart* vs. control games). Decision-making skills as a moderator will be compared between the two study arms at (a) the various time points; (b) change from baseline at various time points and overall study duration; (c) improvement (no/yes change) and (d) longitudinally. We will use t-tests for continuous variables, chi-square for comparing proportion of participants who showed improvement from baseline to each study time point and overall, and longitudinal modeling to include measures of moderators over the whole study duration.

## Results

To date (as of 6/30/23), 380 participants have been enrolled (completing parent/guardian consent, adolescent assent and baseline assessments) and 373 participants have been randomized (7 did not continue with enrollment before randomization occurred). After randomization, three participants withdrew from the study and informed study staff. Currently, 370 participants are enrolled. The following have completed the follow-up protocol defined assessments: post-gameplay: 343 (92.7%); 3 months: 296 (80.0%); 6 months: 223 (60.3%); 12 months: 69 (18.7%). Currently 380 participants are in the assessment follow-up phase of the project, having completed the intervention (gameplay) phase of the project with assessment time windows opening soon for their upcoming assessments, and none actively in the intervention (gameplay) phase as the academic year has come to an end.

Data will be made available through the NIH Helping to End Addiction Long-term (HEAL) Initiative Public Access and Data Sharing Policy that provides guidance on an infrastructure for researchers, clinicians, and patients to share their information and knowledge; our study complies with this policy [73].

## Discussion

The opioid epidemic is a growing critical national crisis. In adolescents, it is estimated that one in seven high school students have misused opioids in their lifetime and one in 14 high school students is currently misusing opioids [15]. Many of the current approaches to addressing opioid misuse in adolescents occur through monitoring and regulating entities that are able to prescribe opioids and statewide policies that limit the number of opioids being prescribed [74]. However, many of these approaches occur after their use has developed into opioid use disorder [16, 75]. Furthermore, prevention programs have been limited, with opioid misuse being rolled into prevention campaigns or school-based interventions having minimal dose, no rigorous evaluation for the efficacy of the program [76] or are not specifically designed for adolescents [77]. Therefore, there is a critical need for prevention-focused efforts and interventions to adequately address the opioid epidemic in this vulnerable population.

Moreover, in the area of digital health games that specifically focus on mental health, there have been some interventions that have been evaluated through randomized controlled trials that focus on specific topics like stress, anxiety and depression, but the *PlaySmart* game is the only intervention that addresses both mental health and opioid misuse [78–82] and recently was recognized as an important resource for youth mental health by the Office of the U.S. Surgeon General [14].

There are potential limitations to this work including our assessments being self-report data; for example, we are not collecting urine samples to test for actual opioid misuse. However, our methods for data collection, that have been used and refined for a number of our prior trials, focus on optimizing privacy and confidentiality for our participants so they are encouraged to respond to questions accurately and honestly. Finally, while the COVID-19 pandemic intermittently introduced challenges in recruitment and data collection, the team was consistently pivoting, using online or virtual means to enroll and record participant responses, when needed. Despite this, we have demonstrated successful recruitment, enrollment, and collection of follow-up data and are very confident that that success will continue until the conclusion of the trial.

The comprehensive and rigorous approach to evaluating the efficacy of *PlaySmart* through the proposed RCT is critical in demonstrating its impact on both preventing opioid misuse and promoting mental health in older adolescents. As a highly accessible and engaging digital health game intervention, *PlaySmart* holds the promise of delivering its evidence-based knowledge/information and skill-building in a manner that has a high likelihood of “sticking” while having the potential of widespread dissemination and extensive reach to adolescent populations.

## Supporting information

S1 Checklist SPIRIT outcomes checklist. (PDF)

S1 Protocol Complete approved institutional review board protocol. (PDF)
