# Supplementary material for: A digital health game to prevent opioid misuse and promote mental health in adolescents in school-based health settings: Protocol for the PlaySmart game randomized controlled trial
Source: PLoS One. 2023 Sep 8;18(9):e0291298. doi: 10.1371/journal.pone.0291298 (PMC10490848; doi:10.1371/journal.pone.0291298)
Supplement: S1 Protocol — (PDF) [file pone.0291298.s002.pdf]

CLINICAL STUDY PROTOCOL

A digital intervention to prevent the initiation of opioid misuse in adolescents in school-based health centers (Randomized Controlled Trial)

Principal Investigator

Lynn E. Fiellin, MD

Sponsor

NIDA HEAL Initiative

Three White Flint North, 11601 Landsdown St, North Bethesda, MD, 20852, United States

https://heal.nih.gov

Protocol Version

10 February 2023

Confidentiality Statement:

Study Personnel

Principal Investigator

Lynn Fiellin
Email: lynn.sullivan@yale.edu

Key Study Personnel:

Tyra Pendergrass
Email: tyra.pendergrass@yale.edu

Claudia-Santi Fernandes
Email: claudiasanti.fernandes@yale.edu

Jennifer Anziano
Email: jennifer.anziano@yale.edu

Francesca Giannattasio
Email: francesca.giannattasio@yale.edu

Lily Hoerner
Email: lily.hoerner@yale.edu

Uche Asuzu
Email:kammarauche.asuzu@yale.edu

Synopsis

Study Purpose

Most opioid misuse begins during adolescence and young adulthood. Adolescence is the time to intervene with prevention interventions (i.e., interventions focused on adolescents who have not yet misused opioids) in settings like school-based health centers (SBHCs), yet few interventions exist that prevent initiation of opioid misuse. "Serious videogame" interventions can improve health behaviors. They meet adolescents "where they are," and compared to standard interventions, they can reach large populations, with consistent fidelity, place limited demands on personnel/resources, and facilitate rapid sustainable distribution, all at a potentially lower cost. This study harnesses the power of videogame interventions and incorporates components of effective substance use prevention programs to develop an evidence-informed intervention to prevent the initiation of opioid misuse in adolescents. Building on our experience developing videogame interventions and in partnership with the national School-Based Health Alliance (SBHA), we developed and will test a new videogame intervention, PlaySmart. PlaySmart was built upon our PlayForward videogame intervention platform that has demonstrated efficacy in improving attitudes and knowledge related to risk behaviors. Through rigorous formative work and with input from adolescents, and our SBHA and game development partners, we have created the PlaySmart videogame intervention. PlaySmart is designed to provide players with behavioral skills and knowledge through repetitive and engaging videogame play to target adolescent perception of risk of harm from initiating opioid misuse. The specific aims of this proposal are to: 1) Conduct a randomized controlled trial (RCT) with 532 high-risk adolescents in 10 SBHCs in CT, to compare PlaySmart to attention/time control games, with assessments at 6 weeks, 3, 6, and 12 months following enrollment to determine if PlaySmart: 1) increases proportion of participants who report a perception of great risk of harm from misuse of opioids at 3 months; and 2) decreases intentions to misuse opioids; 3) increases self-efficacy for refusing opioids; 4) prevents initiation of opioid misuse, at all-time points The gap in the research on preventing initiation of opioid misuse in youth and in implementing prevention programs with good fidelity needs to be urgently addressed given the high prevalence of adolescent opioid misuse and overdose. This research has the potential to create a videogame intervention to prevent initiation of opioid misuse with far-reaching and sustained impact on adolescents.

Primary Objective

Our study's specific aims are to:

1.Conduct a randomized controlled trial with 532 high-risk adolescents in 10 SBHCs, to compare PlaySmart to attention/time control games, with assessments post- gameplay (6 weeks), and at 3, 6, and 12 months following enrollment to determine if PlaySmart: 1) increases proportion of participants who report a perception of great risk of harm from misuse of opioids at 3 months; and 2) decreases intentions to misuse opioids; 3)

increases self-efficacy for refusing opioids; 4) prevents initiation of opioid misuse, at all time-points.

Secondary Objectives (if applicable)

N/A

General Design Description

We will conduct a randomized controlled trial in 10 of our partner SBHCs, enrolling 532 high-risk adolescents. All participants will be provided with substance use prevention information from a pamphlet from NIDA and then randomized to play either the experimental game PlaySmart or a menu of commercial, off-the-shelf educational and entertaining games devoid of any relevant content. The primary outcome will be the proportion of participants who report great risk of harm from use of opioids (prescription opioids and heroin/fentanyl) at 3 months. We will test for durability of the intervention effect by collecting data at 6 and 12 months.

We will also conduct a process evaluation as part of the RCT (Implementation/RCT), conducting interviews and surveys with participants who played PlaySmart and two SBHC "champions" at each of the 10 RCT sites at a specified timepoint. Data will also be collected from the gameplay software (in-game data) and analyzed using a system we have developed for our other videogame interventions.

Study Date Range and Duration

The probable duration of the project is 3 years. This timeline includes recruitment/ enrollment in the randomized controlled trial, up to 6 hours of gameplay sessions, and baseline, post-gameplay and follow-up assessments at 3-, 6- and 12-months after enrollment for 532 participants. The study will begin in September 2021.

Number of Study Sites

10 high schools with school-based health centers in Connecticut.

Primary Outcome Variables

The primary outcome will be the proportion of participants who report great risk of harm from use of opioids (prescription opioids and heroin/fentanyl) at 3 months. We will test for durability of the intervention effect by collecting data at 6 and 12 months.

Secondary and Exploratory Outcome Variables (if applicable)

PlaySmart will aim to prevent initiation of opioid misuse by influencing important theoretical antecedents and/or mediators/moderators including 1) intentions to misuse opioids; 2) self-efficacy to refuse offers of opioids; 3) attitudes about opioid misuse; 4) knowledge about opioid misuse and its risk; 5) perceived norms about opioid misuse; and 6) decision-making skills.

**Study Population**<br>The subject population for this project will be 532 at-risk adolescents between the ages of 16-19. Adolescent participants must: 1) attend a high school that preferably has a SBHC and preferably be enrolled in the school's school based health center; 2). be 16-19 years (grades 9-12); 3) report not having engaged in any prior opioid misuse; 4) be at "high-risk" based on their report at baseline of past 30-day use of cigarettes, e-cigarettes, Juul, alcohol, marijuana (including synthetics), amphetamine, cocaine, benzodiazepines, ecstasy, bath salts, or any other misuse of non-opioid prescription drugs or use of non-opioid illicit drugs (44,45) OR have a score of ≥ 1 on the modified PHQ-2 (149) OR a score of ≥ 1 on the modified GAD-2 (150) (both screening tools used by SBHA); 5) be willing to sit for 60 minutes/session to play the game; and 6) be able to provide assent/parental/guardian consent (if under age 18). These adolescents will be recruited from 10 high schools with SBHCs in Connecticut where our team has developed partnerships. Our team has a successful track record of establishing a consistent presence at our partner sites and now has close working relationships with the program staff that are fully engaged in the recruitment process. Sites for recruitment and participation include but are not limited to: Hamden High School, Francis T. Maloney High School, Platt High School and Stratford High School.<br><br>We will also engage 20 school-based health center personnel that assisted with the randomized controlled trial. We will conduct a process evaluation at the 10 RCT sites to assess implementation. We will collect qualitative and quantitative data from each of the 10 RCT sites from participants who played PlaySmart and SBHC personnel through semi-structured interviews and surveys every three months and through in-game data.
**Number of Participants**<br>532 high-risk adolescents<br><br>20 school-based health center staff
**Visit Schedule Table (0ptional)**<br>• Study activities will include: 1) playing an iPad or sitting at a computer to play an original game developed by the play2PREVENT Lab or a set of control games 2) answering questions about experience playing the game, and thoughts, attitudes and knowledge around a health topic.<br>• Involvement will require: 1) 60 minutes of time during or after school, one to two times per week, for up to 6 weeks (six sessions total); 2) completing assessment questions (that take approximately 20-30 mins to complete) at different time points associated with the study (baseline, post-gameplay [6 weeks], 3 months, 6 months and 12 months)
**Study Flow Chart (optional)**

N/A

Abbreviations

Abbreviation	Explanation

Glossary of Terms

Glossary	Explanation

Table of Contents

Synopsis

Study Purpose

Primary Objective

Secondary Objectives (if applicable)

General Design Description

Study Date Range and Duration

Number of Study Sites

Primary Outcome Variables

Secondary and Exploratory Outcome Variables (if applicable)

Study Population

Number of Participants

Visit Schedule Table (0ptional)

Study Flow Chart (optional)

Abbreviations

Glossary of Terms

1.1 Statement of Compliance

2.1 Background

3.1 Problem Statement

3.2 Purpose of Study/Potential Impact

3.3.1 Potential Risks

3.3.2 Potential Benefits

4.1 Hypothesis

4.2 Primary Objective

4.3 Secondary Objectives (if applicable)

5.1 General Design Description

5.1.1 Study Date Range and Duration

5.1.2 Number of Study Sites

5.2 Outcome Variables

5.2.1 Primary Outcome Variables

5.2.2 Secondary and Exploratory Outcome Variables (if applicable)

5.3 Study Population ....

5.3.1 Number of Participants ....

5.3.2 Eligibility Criteria/Vulnerable Populations ....

6.1 Intervention ....

6.1.1 Description of Intervention ....

6.1.2 Method of Assignment/Randomization ....

6.1.3 Selection of Instruments/Outcome Measures ....

6.1.4 Intervention Administration ....

6.1.5 Reaction Management ....

6.2 Assessments ....

6.2.1 Efficacy ....

6.2.2 Safety/Pregnancy-related Procedure ....

6.2.3 Adverse Events Definition and Reporting ....

6.2.4 Pharmacokinetics (if applicable) ....

6.2.5 Biomarkers (if applicable) ....

6.3 Study Procedures ....

6.3.1 Study Schedule ....

6.3.2 Informed Consent ....

6.3.3 Screening ....

6.3.4 Recruitment, Enrollment and Retention ....

6.3.5 Study Visits ....

6.3.6 End of Study and Follow Up ....

6.3.7 Removal of Subjects ....

6.4 Statistical Method ....

6.4.1 Statistical Design ....

6.4.2 Sample Size Considerations ....

6.4.3 Planned Analyses ....

6.4.3.1 Primary Analyses ....

6.4.3.2 Secondary Objectives Analyses, if applicable ....

6.4.3.3 Analysis of Subject Characteristics ....

6.4.3.4 Interim Analysis (if applicable) ....

6.4.3.5 Health economic evaluation, if applicable

6.4.3.6 Other

6.4.4 Subsets and Covariates

6.4.5 Handling of Missing Data

7.1 Ethical Considerations: Informed Consent/Assent and HIPAA Authorization

7.2 Institutional Review Board (IRB) Review

7.3 Subject Privacy, Confidentiality & Data Management

7.4 Deviations/Unanticipated Problems

7.5 Data Collection

7.6 Data Quality Assurance

7.7 Study Records

7.8 Access to Source

7.9 Data or Specimen Storage/Security

7.10 Retention of Records

7.11 Study Monitoring

7.12 Data Safety Monitoring Plan

7.13 Study Modification

7.14 Study Discontinuation

7.15 Study Completion

7.16 Conflict of Interest Management Plan

7.17 Funding Source

7.18 Publication Plan

Appendices

List of Tables

References

1.1 Statement of Compliance

This document is a protocol for a human research study. The purpose of this protocol is to ensure that this study is to be conducted according to the Common Rule at 45CFR46 (human subjects) and other applicable government regulations and Institutional research policies and procedures.

2.1 Background

Drug overdose is now the leading cause of death in those under the age of 50. According to the Centers for Disease Control and Prevention (CDC), 130 Americans die every day from an opioid overdose (1) . These overdose mortality rates reflect the increase of opioid misuse in this country and of particular concern, is that they translate to adolescents. Opioid overdose deaths among adolescents aged 15—19 increased 253% between 1999 and 2016 (2) . Importantly, close to 40% of prescription opioid misuse and 68% of heroin use starts in adolescence and young adulthood3.

Given the importance of this problem to the health and future of our youth, it is imperative to develop and implement effective strategies to prevent the initiation of opioid misuse in youth. The current situation demands high quality, evidence-informed programs that can be rapidly deployed with good fidelity. To date, programs created to prevent substance use initiation overall (e.g., alcohol, marijuana, and tobacco) do not have much impact in terms of effectively preventing initiation of opioid misuse. Further, there is conflicting evidence on the effectiveness of substance use prevention interventions, particularly those conducted in schools where many of these programs are deployed. Novel ways to reach this highly vulnerable population in settings such as the over 2600 SBHCs are needed.

Videogames as interventions have the advantage of meeting adolescents "where they are." Over 90% of adolescent boys and girls play videogames (3) . "Serious games," defined as games for a primary purpose other than entertainment, have been developed by our group and others and there is compelling evidence that they promote health and are effective at prevention (4) (5) (6). Serious videogames are an ideal prevention platform, facilitating opportunities for repetitive interactions to acquire and rehearse new skills that can transfer to real-life situations (7) (8). They can reach large populations, with consistent fidelity, place limited demands on personnel/resources, and facilitate rapid sustainable distribution, all at a potentially lower cost. To date, no effective serious videogame interventions to prevent initiation of opioid misuse have been developed. While gaming addiction is a recognized entity, it is very rare and, in the setting of playing a serious game, very unlikely. If there are any concerns of developing serious addictive behaviors in our study participants, we have team members who are experts in the field of addiction medicine and will address these issues immediately. We built on our experience developing and evaluating serious videogame interventions (R01 HD062080; R41 HD088317, 2R42 HD088317, 5P50 DA036151) (6-9,12-26) to develop a videogame intervention designed to prevent initiation of opioid misuse in adolescents. We conducted a formative work with our target audience and content experts to create a new videogame intervention, PlaySmart, focusing on preventing initiation of opioid misuse in older adolescents, aged 16-19. Content experts include those from building out the foundation of PlayForward: Elm City Stories that targets youth HIV prevention (5) and PlayForward: smokeSCREEN that targets youth smoking prevention (6) . We built the conceptual model19 to promote healthy decision-making using constructs from the theory of planned behavior (9), social learning and self- efficacy theories (10) and principles from message framing (11) grounded in prospect theory that have been shown to impact prevention. We focused on antecedents of opioid misuse including perceived harm, intentions, self-efficacy, and initiation of opioid misuse. This study is consistent with

objectives outlined in the proposed National Institute on Drug Abuse 2016-2020 Strategic Plan in that it will "develop and test innovative prevention interventions that target mechanisms underlying risk factors" exploring the "potential of technology- based methods for delivering prevention interventions, such as smartphones, video games, and social media." It also focuses on the "vulnerability for substance use and related problems…shown to peak during critical life transitions (12) " as seen in high-risk adolescents.

Our study's specific aims are to:

1.Conduct a randomized controlled trial with 532 high-risk adolescents in 10 SBHCs, to compare PlaySmart to attention/time control games, with assessments post- gameplay (6 weeks), and at 3, 6, and 12 months following enrollment to determine if PlaySmart: 1) increases proportion of participants who report a perception of great risk of harm from misuse of opioids at 3 months; and 2) decreases intentions to misuse opioids; 3) increases self-efficacy for refusing opioids; 4) prevents initiation of opioid misuse, at all time-points.School-based health center personnel will assist with the recruitment, screening and enrollment of participants in the randomized controlled trial.

Evaluate implementation strategies with school-based health center personnel through quantitative and qualitative data.

3.1 Problem Statement

Drug overdose is now the leading cause of death in those under the age of 50. According to the Centers for Disease Control and Prevention (CDC), 130 Americans die every day from an opioid overdose (1). These overdose mortality rates reflect the increase of opioid misuse in this country and of particular concern, is that they translate to adolescents. Opioid overdose deaths among adolescents aged 15—19 increased 253% between 1999 and 2016(2). Importantly, close to 40% of prescription opioid misuse and 68% of heroin use starts in adolescence and young adulthood (3).

Given the importance of this problem to the health and future of our youth, it is imperative to develop and implement effective strategies to prevent the initiation of opioid misuse in youth. The current situation demands high quality, evidence-informed programs that can be rapidly deployed with good fidelity. To date, programs created to prevent substance use initiation overall (e.g., alcohol, marijuana, and tobacco) do not have much impact in terms of effectively preventing initiation of opioid misuse. Further, there is conflicting evidence on the effectiveness of substance use prevention interventions, particularly those conducted in schools where many of these programs are deployed (4). Novel ways to reach this highly vulnerable population in settings such as the over 2600 SBHCs are needed.

Videogames as interventions have the advantage of meeting adolescents "where they are." Over 90% of adolescent boys and girls play videogames (5). "Serious games," defined as

games for a primary purpose other than entertainment, have been developed by our group and others and there is compelling evidence that they promote health and are effective at prevention (6-9). Serious videogames are an ideal prevention platform, facilitating opportunities for repetitive interactions to acquire and rehearse new skills that can transfer to real-life situations(10,11). They can reach large populations, with consistent fidelity, place limited demands on personnel/resources, and facilitate rapid sustainable distribution, all at a potentially lower cost. To date, no effective serious videogame interventions to prevent initiation of opioid misuse have been developed. While gaming addiction is a recognized entity, it is very rare and, in the setting of playing a serious game, very unlikely. If there are any concerns of developing serious addictive behaviors in our study participants, we have team members who are experts in the field of addiction medicine and will address these issues immediately. We built on our experience developing and evaluating serious videogame interventions (R01 HD062080; R41 HD088317, 2R42 HD088317, 5P50 DA036151) (6-9,12-26) to develop a videogame intervention designed to prevent initiation of opioid misuse in adolescents. We conducted a formative work with our target audience and content experts to create a new videogame intervention, PlaySmart, focusing on preventing initiation of opioid misuse in older adolescents, aged 16-19. Content experts include those from building out the foundation of PlayForward: Elm City Stories that targets youth HIV prevention (7) and PlayForward: smokeSCREEN that targets youth smoking prevention8. We built the conceptual model (19) to promote healthy decision-making using constructs from the theory of planned behavior (27), social learning and self- efficacy theories (28,29) and principles from message framing (30) grounded in prospect theory (31) that have been shown to impact prevention. We focused on antecedents of opioid misuse including perceived harm, intentions, self-efficacy, and initiation of opioid misuse. This study is consistent with objectives outlined in the proposed National Institute on Drug Abuse 2016-2020 Strategic Plan in that it will "develop and test innovative prevention interventions that target mechanisms underlying risk factors" exploring the "potential of technology- based methods for delivering prevention interventions, such as smartphones, video games, and social media." It also focuses on the "vulnerability for substance use and related problems…shown to peak during critical life transitions(32)" as seen in high-risk adolescents.

Our study's specific aims are to:

1.Conduct a randomized controlled trial with 532 high-risk adolescents in 10 SBHCs, to compare PlaySmart to attention/time control games, with assessments post- gameplay (6 weeks), and at 3, 6, and 12 months following enrollment to determine if PlaySmart: 1) increases proportion of participants who report a perception of great risk of harm from misuse of opioids at 3 months; and 2) decreases intentions to misuse opioids; 3) increases self-efficacy for refusing opioids; 4) prevents initiation of opioid misuse, at all time-points.

We will conduct a process evaluation at the 10 RCT sites to assess implementation. We will use formal instruments to measure fidelity, intervention “dose” delivered, “dose” received, and participant and SBHC personnel satisfaction, context, and reach. We will collect

qualitative and quantitative data from each of the 10 RCT sites from participants who played PlaySmart and SBHC personnel through semi-structured interviews and surveys every three months and through in-game data. Data will be collected on the following factors: (1) Fidelity: Proportion of participants who completed all gameplay; (2) Dose delivered: Time per session and overall that gameplay was available; (3) Dose received: Time per session and overall of participant gameplay; (4) Participant and SBHC personnel gameplay satisfaction: The extent to which participants and SBHC personnel enjoyed the game intervention, including using open-ended questions; (5) Reach: Proportion of participants with any gameplay; and (6) Context: Barriers and facilitators to implementing the game intervention.

3.2 Purpose of Study/Potential Impact

There is a dire need to prevent the initiation of opioid misuse in adolescents given the high prevalence of misuse and overdose. Completion of this study will produce a rigorously developed and tested, theory informed videogame intervention targeting perceived risk of harm from opioids in older adolescents. We chose to target perception of risk of harm from drug use as the primary outcome given its clear inverse relationship with actual drug use. We built the game on appropriate theoretical foundations and include components of established prevention strategies. The scientific premise behind this study and the evidence for this type of novel intervention are strong. This study fills an essential gap in the research on preventing initiation of opioid misuse in adolescents by creating a tailored game for older adolescents, and by incorporating the key features of effective substance use prevention interventions into a new delivery vehicle that has demonstrated potential to influence health, including risk for opioid misuse, and can be implemented with limited resources, with fidelity, and at a potentially lower cost. With the wide-spread reach of the national SBHA and with the delivery vehicle of a videogame, this project has the potential to address the challenges of reach/access, implementation, fidelity, personnel, sustainability, and cost.

3.3.1 Potential Risks

The potential risks associated with this study have to do with maintenance of the confidentiality of the identities of the participants enrolled in the study and information relating to them. De-identified hard copies of the data will be stored in locked cabinets and electronic data will be stored in secure, password-protected computers. Only the PI and other relevant research staff will have access to the data.

Playing the ***PlaySmart*** game or engagement in discussions with the research team about its content may pose a potential psychological risk in that we address sensitive issues around drug use and its consequences. The research staff will be available at all times to provide assistance to all participants, answer their questions, and serve as a resource if any distress or concern arises. The assessments and instruments may also present a potential risk given that some of the questions are sensitive in nature and address issues around drug use risky behaviors. Again, the research staff will be available to assist the participants and the program site staff if there appears to be any distress around the questions being asked.

If there are concerns about issues raised with regards to substance use or mental health, or if participants need additional or more intensive attention, the research staff will contact Dr. L. Fiellin or Dr. Fernandes (who is a Licensed Professional Counselor and has extensive expertise working with adolescents and schools) who will discuss providing further consultation to the participant.

3.3.2 Potential Benefits

It is anticipated that the adolescents enrolled in this study should benefit directly from the study as the purpose of the study is to facilitate increasing their perception of risk of harm from initiating the misuse of opioids, decreasing their actual misuse of opioids, as well as improving their decision making and knowledge to optimize their abilities to negotiate around risky behaviors. The potential benefits to participants are great while strict precautions are being taken to protect the confidentiality and well-being of participants, as has been described above. Thus the potential benefits to the participants outweigh the potential risks.

4.1 Hypothesis

The primary hypothesis is that, at 3 months, there will be a higher proportion of PlaySmart vs. control participants who report greater risk of harm from misuse of prescription opioids AND heroin/fentanyl.

4.2 Primary Objective

Our study's specific aims are to:

1.Conduct a randomized controlled trial with 532 high-risk adolescents in 10 SBHCs, to compare PlaySmart to attention/time control games, with assessments post- gameplay (6 weeks), and at 3, 6, and 12 months following enrollment to determine if PlaySmart: 1) increases proportion of participants who report a perception of great risk of harm from misuse of opioids at 3 months; and 2) decreases intentions to misuse opioids; 3) increases self-efficacy for refusing opioids; 4) prevents initiation of opioid misuse, at all time-points.

4.3 Secondary Objectives (if applicable)

N/A

5.1 General Design Description

PlaySmart is a web-based, interactive videogame. Based on the design of components in ***PlayForward***, in ***PlaySmart***, the player will create an *Aspirational Avatar*, a character that represents their future hopes and dreams, with an emphasis on aspirations related to their overall health and well-being. The player will then guide their avatar through an overarching graphic novel-like story about navigating risks as

a teenager, including those associated with opioid and other substance use. To advance in the game, the player must acquire "skill points" by playing through a series of smaller, skill-based mini-games that are embedded within the larger gameplay experience. Each mini-game will focus on increasing one of the specific skills or attributes that have been identified as effective components of substance use prevention interventions (see section A.5). The mini-games will focus on 1) increasing perceived risk of harm from initiating misuse of opioids, with additional focus on the risk of initiating any substance use; 2) reframing cognitive distortions; 3) refusing offers to misuse opioids; 4) acquiring knowledge about opioid misuse as well as about the social influences and risk factors that lead to opioid misuse; 5) spreading opioid misuse awareness; and 6) improving sense of future orientation. Building on these skills and attributes should ultimately lead to increased perception of risk of harm from opioid misuse with the goal of preventing initiation. The game also highlights hoe to find help for mental health challenges that can co-occur with substance misuse and includes positive messages throughout the storylines, such as the positive aspects of staying healthy and accomplishing other goals in their life.

Sample Mini-games: Mini-games are focused on specific constructs related to behavior change, and increasing levels of mastery are required to progress the story. Sample mini-games include: ***Risk Sense:*** In this mini-game, the player becomes aware of numerous risk factors for opioid misuse and takes steps to prevent risks from harming the character. ***Stress Sense:*** This mini-game models how identify a cognitive distortion and then chose to reframe it or learn about coping skills. ***Refusal Power:*** In this mini-game, the player practices refusing offers to misuse opioids from their peers in high pressure situations. ***Social Media Game:*** In this mini-game, the player creates positively-framed memes or posters about the health benefits of avoiding opioids to share with their peers in the game with the goal of influencing their attitudes and intentions. ***Know Power***: In this game, players engage in a series of group text-message conversations with their friends to discuss critical facts about opioids and opioid misuse.

We will conduct a randomized controlled trial with 10 of our partner SBHCs, enrolling 532 high-risk adolescents. All participants will be provided with substance use prevention information from a pamphlet from NIDA and then randomized to play either the experimental game *PlaySmart* or a menu of commercial, off-the-shelf educational and entertaining games

devoid of any relevant content. The primary outcome will be the proportion of participants who report great risk of harm from use of opioids (prescription opioids and heroin/fentanyl) at 3 months. We will test for durability of the intervention effect by collecting data at 6 and 12 months.

We will also conduct a process evaluation as part of the RCT (Implementation/RCT), conducting interviews and surveys with participants who played PlaySmart and two SBHC "champions" at each of the 10 RCT sites at a specified timepoint. Data will also be collected from the gameplay software (in-game data) and analyzed using a system we have developed for our other videogame interventions.

5.1.1 Study Date Range and Duration

The probable duration of the project is 3 years. This timeline includes recruitment/enrollment in the randomized controlled trial, up to 6 hours of gameplay sessions, and baseline, post-gameplay and follow-up assessments at 3-, 6- and 12-months after enrollment for 532 participants. The study will begin in September 2021.

5.1.2 Number of Study Sites

10 high schools with school-based health centers in Connecticut.

5.2 Outcome Variables

5.2.1 Primary Outcome Variables

The primary outcome will be the proportion of participants who report great risk of harm from use of opioids (prescription opioids and heroin/fentanyl) at 3 months. We will test for durability of the intervention effect by collecting data at 6 and 12 months.

5.2.2 Secondary and Exploratory Outcome Variables (if applicable)

1) intentions to misuse opioids; 2) self-efficacy for refusing opioids; 3) knowledge about opioids and opioid misuse; 4) beliefs and attitudes around opioid misuse; 5) initiation of opioid misuse, at all time-points.

5.3 Study Population

The subject population for this project will be 532 at-risk adolescents between the ages of 16-19. Adolescent participants must: 1) attend a high school that preferably has a SBHC and preferably be enrolled in the school based health center; 2). be 16-19 years (grades 9-12); 3) report not having engaged in any prior opioid misuse; 4) be at "high-risk" based on their report at baseline of past 30-day use of cigarettes, e-cigarettes, Juul, alcohol, marijuana (including synthetics), amphetamine, cocaine, benzodiazepines, ecstasy, bath salts, or any other misuse of non-opioid prescription drugs or use of non-opioid illicit drugs (44,45) OR have a score of ≥ 1 on the modified PHQ-2 (149) OR a score of ≥ 1 on the modified GAD-2 (150) (both screening tools used by SBHA); 5) be willing to sit for 60 minutes/session to play the game; and 6) be able to provide assent/parental/guardian consent (if under age 18). These adolescents will be recruited from 10 high schools with SBHCs in Connecticut where our team has developed partnerships. Our team has a successful track record of establishing a consistent presence at our partner sites and now has close working relationships with the program staff that are fully engaged in the recruitment process. Sites

for recruitment and participation include but are not limited to: Hamden High School, Francis T. Maloney High School, Platt High School and Stratford High School.

In addition, we will also engage twenty (2 “personnel” or champions from each of th 10 sites) school based health center staff (aged 18-65) at our partner high schools and have them fill out survey question to gain their input on their experience with the randomized controlled trial and ways the game could be implemented outside of the randomized controlled trial.

5.3.1 Number of Participants

532 high-risk adolescents

20 school-based health center staff

5.3.2 Eligibility Criteria/Vulnerable Populations

Adolescent participants must: 1) attend a high school that preferably has a school-based health center and preferably be enrolled in the school’s school-based health center 2). be 16-19 years (grades 9-12); 3) report <u>not</u> having engaged in any prior opioid misuse; 4) be at "high-risk" based on their report at baseline of past 30-day use of cigarettes, e-cigarettes, Juul, alcohol, marijuana (including synthetics), amphetamine, cocaine, benzodiazepines, ecstasy, bath salts, or any other misuse of non-opioid prescription drugs or use of non-opioid illicit drugs (44,45) <u>OR</u> have a score of ≥ 1 on the modified PHQ-2 (149) <u>OR</u> a score of ≥ 1 on the modified GAD-2 (150) (both screening tools used by SBHA); 5) be willing to sit for 60 minutes/session to play the game; 6) be able to provide assent/parental/guardian consent (if under age 18

Adult participants ("champions") must be personnel of a SBHC where we are conducting the RCT and must have assisted the research team in recruitment, screening and enrollment of participants in the randomized controlled trial.

6.1 Intervention

6.1.1 Description of Intervention

PlaySmart was built upon our PlayForward videogame intervention platform that has demonstrated efficacy in improving attitudes and knowledge related to risk behaviors. Through rigorous formative work and with input from adolescents, and our SBHA and game development partners, we have created the PlaySmart videogame intervention. PlaySmart is designed to provide players with behavioral skills and knowledge through repetitive and engaging videogame play to target adolescent perception of risk of harm from initiating opioid misuse.

6.1.2 Method of Assignment/Randomization

We will use a stratified randomization procedure to assign participants to one of the two study arms. Gender and grade (9/10th and 11/12th) will be included as stratification variables in the randomization algorithm because these variables have been found to have important associations with initiation of drug use in adolescents and young adults.

6.1.3 Selection of Instruments/Outcome Measures

(Please see attached sheet for a chart of instruments that will be used in this study)

6.1.4 Intervention Administration

We will enroll 532 high-risk girls and boys who attend one of our 10 partner schools. Eligible individuals will be assigned to either the (1) PlaySmart arm (n=266) or (2) a attention/time control games arm (n=266). Both groups will receive a pamphlet developed by NIDA on "Opioids Facts for Teens." This includes information on frequently asked questions, prescription opioids, fentanyl and heroin, and other useful resources. All participants will be assigned to play up to two sessions per week of their assigned game(s) for six weeks for a goal of 6 hours of gameplay. All gameplay sessions will either occur on site at their schools or virtually through a selected platform where the research team can observe the students' screens during the game. These session will be done in collaboration with the SBHC and its personnel ("champions") who will have been oriented to the game implementation manual. This total duration and number of sessions is consistent with those found in effective substance use prevention interventions, with the amount of time adolescents play games, and the amount of gameplay observed among participants in our RCT of PlayForward. We will collect follow-up data on all participants at six weeks (immediately following completion of gameplay), three, six, and 12 months. Students who are randomized to expiermental will also have the opportunity to participate in recorded qualitative interviews about their gameplay expierence following completion of the gameplay, if the box on both their parental consent and adolescent assent has been checked indicating they have consent to participate in the recorded portion.

6.1.5 Reaction Management

If there are concerns about issues raised with regards to substance use or mental health, or if participants need additional or more intensive attention, the research staff will contact Dr. L. Fiellin or Dr. Fernandes (who is a Licensed Professional Counselor and has extensive expertise working with adolescents and schools) who will discuss providing further consultation to the participant.

6.2 Assessments

6.2.1 Efficacy

(Please see attached document that has the measures that will be utilized in this study to test the efficacy of the videogame intervention, PlaySmart).

6.2.2 Safety/Pregnancy-related Procedure

The PI will be responsible for monitoring the data and conducting performance and safety reviews, at the specified frequency. Either the PI or the IRB have the authority to stop or modify the study. The monitoring by the IRB will occur annually at the time of re-approval. The PI will conduct data and safety review at least quarterly and at any time a serious adverse event occurs. During the review process, the PI will evaluate whether the study should continue unchanged, requires modification or amendment to continue, or should be closed to enrollment.

6.2.3 Adverse Events Definition and Reporting

This study presents minimal risks to the participants and adverse events or other problems are not anticipated.

Adverse Events (AEs):

Violation of confidentiality: Any breach of confidentiality.

Discomfort due to assessment procedures: Discomfort created by answering survey questions.

Embarrassment in disclosing sensitive personal information.

Disclosure of information about current and/or intended physical harm to persons; current and/or intended abuse of children that would be reported to a child welfare agency; and/or an investigation of such allegations(s) that could ensue.

Dissatisfaction with the intervention activities: In this case it would be the game intervention.

Serious Adverse Events (SAEs):

SAEs include death, life threatening injury or condition, hospitalization, persistent or significant disability/incapacity, and other conditions which in the judgment of the investigators represent significant hazards.

Procedures for research team management of AEs, SAEs and other study risks such as mandatory reporting requirements:

This study presents minimal risks to the participants and adverse events or other problems are not anticipated. In the unlikely event that such events occur, Reportable Adverse Events (which are events that are serious or life-threatening and unanticipated (or anticipated but occurring with a greater frequency than expected) and possibly, probably, or definitely related) or unanticipated problems involving risks to subjects or others will be reported in writing within 24 hours to the IRB (using the appropriate forms from the website) and the NIDA PO with a follow-up report within 72 hours. The PI will apprise fellow investigators and study personnel of all adverse events that occur during the conduct of this research project through regular study meetings and via email as they are reviewed by the PI. We will use procedures to detect and respond to adverse events that ensure prompt discovery of any adverse events and to minimize their effects. There is adequate surveillance and protections to discover adverse events promptly and keep their effects minimal.

Procedures for training and supervision of all research personnel have been developed as part of our previous and current studies. All members of the research team are familiar with procedures for identifying and reporting possible adverse reactions. All members of the

research team are trained in mandatory reporting and will notify the PI immediately of any concerns raised about participant mental health (suicidality), substance use, abuse of any kind, and so forth.

If an adverse event occurs study personnel will notify the PI as well as the appropriate Information Security Officer (ISO) and follow all necessary procedures. All adverse events are reported using the Yale Institutional Review Board (IRB) standard template for reporting adverse events. The PI reviews all adverse events, classifies the attribution of adverse events (e.g., definitely, probably, possibly related; unlikely or unrelated) and grades the severity of the event, utilizing the FDA's definition of serious adverse events, on a 6-point scale (0=no adverse event or within normal limit; 1=mild; 2=moderate; 3=severe; 4=life-threatening; 5=fatal). Serious unanticipated or anticipated adverse events will be reported immediately to the IRB and to the NIDA PO within 24 hours by phone and/or email and will submit a written report to the PO no more than two days later. Adverse events will be reported in summary form at least annually to the IRB. The summary will include the number of participants enrolled and a summary of graded adverse events to date, using the chart format included in the Yale University DSMP template. The PI will evaluate all adverse events and determine whether the event affects the Risk/Benefit ratio of the study and whether modifications to the protocol (e.g., Risks to Subjects) or consent form (e.g., Risks and Inconveniences) are required.

The PI will be responsible for monitoring the data and conducting performance and safety reviews, at the specified frequency. Either the PI or the IRB have the authority to stop or modify the study. The monitoring by the IRB will occur annually at the time of re-approval. The PI will conduct data and safety review at least quarterly and at any time a serious adverse event occurs. During the review process, the PI will evaluate whether the study should continue unchanged, requires modification or amendment to continue, or should be closed to enrollment.

6.2.4 Pharmacokinetics (if applicable)

6.2.5 Biomarkers (if applicable)

6.3 Study Procedures

6.3.1 Study Schedule

We will enroll 532 high-risk girls and boys who attend one of our 10 partner schools. Eligible individuals will be assigned to either the (1) PlaySmart arm (n=266) or (2) a attention/time control games arm (n=266). Both groups will receive a pamphlet developed by NIDA on "Opioids Facts for Teens." This includes information on frequently asked questions, prescription opioids, fentanyl and heroin, and other useful resources. All participants will be assigned to play up to two sessions per week of their assigned game(s) for six weeks for a goal of 6 hours of gameplay. All gameplay sessions will either occur on site at their schools or virtually through a selected platform where the research team can observe the students' screens during the game. These session will be done in collaboration with the SBHC and its personnel ("champions") who will have been oriented to the game implementation manual.. This total duration and number of sessions is consistent with those found in effective

substance use prevention interventions, with the amount of time adolescents play games, and the amount of gameplay observed among participants in our RCT of PlayForward. We will collect follow-up data on all participants at six weeks (immediately following completion of gameplay), three, six, and 12 months.

6.3.2 Informed Consent

Screening and consent:

A member of the research team (in collaboration with the designated school liaison/champion) will screen for eligibility, discuss the study with the participant and their parent/guardian, and obtain written informed assent/electronic informed assent from the individual and written informed consent/electronic informed consent from their parent/guardian (if under age 18). Participants will be provided with an age-appropriate description of the study as "finding out how teens can make choices that are healthier for them." In describing the study to the parent/guardian, we will indicate that the study is about promoting healthy behaviors and on providing strategies to assist adolescents in making decisions about the challenges they face, including the risk for initiating drug use. If an individual or their parent/guardian indicates that they do not wish to participate, there will be no further involvement in the study. In the case of those participants who are over the age of 18 (18-19 years old) or are emancipated minors, we will obtain written informed consent/electronic informed consent from them.

Given the proposed study includes adolescent youth, we will be required in situations such as this to report any concerns that arise at the school as part of required mandatory reporting (all members of the research team have completed mandatory reporter training). This includes mandatory reporting for abuse and neglect to appropriate officials via Careline at 1-800-842-2288. Suicidal risk will be reported to school official as well as parent/guardian. In extreme cases, 9-1-1 will be called.

Fortunately, we will be working closely with the personnel of the school-based health center who are well-trained in managing these types of situations. We will employ all necessary measures for protecting the confidentiality of our participants who agree to be in this pilot study.

This research does not involve subjects with limited decision-making capacity. Parents and adolescents will be provided with an age-appropriate description of the study and told about what they might expect. In describing the study to the participant, the purpose of the study will be described as "finding out how kids can make choices that are healthier for them". In describing the study to the guardian/parent, we will indicate that the study will focus on promoting healthy behaviors in adolescence and on providing strategies to assist adolescents in making decisions about the many challenges they face, including risk for initiating drug use. Parents who do not wish for their children to participate may contact the project director. We will obtain informed written informed assent/electronic informed assent from the participant and written informed consent/electronic informed consent from their guardian/parent.

6.3.3 Screening

A member of the research team (in collaboration with the designated school liaison/champion) will screen for eligibility, discuss the study with the participant and their parent/guardian, and obtain written informed assent/electronic informed assent from the individual and written informed consent/electronic informed consent from their parent/guardian (if under age 18). Participants will be provided with an age-appropriate description of the study as "finding out how teens can make choices that are healthier for them." In describing the study to the parent/guardian, we will indicate that the study is about promoting healthy behaviors and on providing strategies to assist adolescents in making decisions about the challenges they face, including the risk for initiating drug use. If an individual or their parent/guardian indicates that they do not wish to participate, there will be no further involvement in the study. In the case of those participants who are over the age of 18 (18-19 years old) or are emancipated minors, we will obtain written informed consent/electronic informed consent from them.

Given the proposed study includes adolescent youth, we will be required in situations such as this to report any concerns that arise at the school as part of required mandatory reporting (all members of the research team have completed mandatory reporter training). This includes mandatory reporting for abuse and neglect to appropriate officials via Careline at 1-800-842-2288. Suicidal risk will be reported to school official as well as parent/guardian. In extreme cases, 9-1-1 will be called.

Fortunately, we will be working closely with the personnel of the school-based health center who are well-trained in managing these types of situations. We will employ all necessary measures for protecting the confidentiality of our participants who agree to be in this pilot study.

6.3.4 Recruitment, Enrollment and Retention

Participation in this study will be strictly voluntary, confidential and non-discriminatory. Participants will be enrolled while they are on-site at one of the participating schools or either remotely after their after eligibility has been determined and their e-consents and assents are signed. The study will be advertised in myriad ways including: posters, flyers, sign-ups through afterschool programs, open houses, announcements and informational table set up during lunch waves, open houses and report card nights. Announcements will also be made during health classes, and mass emails will be sent out to parents/guardians. The research staff will work in partnership with the SBHC staff to recruit participants. In addition, the SBHC staff will help to screen participants for prior substance use, depression and anxiety. Participants will be provided with an age-appropriate description of the study and told about what they might expect. In describing the study to the participant, the purpose of the study will be described as "finding out how kids can make choices that are healthier for them". In describing the study to the guardian/parent, we will indicate that the study will focus on promoting healthy behaviors in adolescence and on providing strategies to assist adolescents in making decisions about the many challenges they face. If a participant or their guardian/parent indicates that they do not wish to participate, there will be no further

involvement in the study. We will also obtain agreement from the appropriate administrator at the participating program.

6.3.5 Study Visits

- Study activities will include: 1) playing an iPad or sitting at a computer to play an original game developed by the play2PREVENT Lab, 2) answering questions about experience playing the game, and thoughts, attitudes and knowledge around a health topic.
- Involvement will require: 1) 60 minutes of time during or after school, one to two times per week, for up to 6 weeks (six sessions total); 2) completing assessment questions (that take approximately 20-30 mins to complete) at different time points associated with the study (baseline, post-gameplay [6 weeks], 3 months, 6 months and 12 months)

6.3.6 End of Study and Follow Up

End of Study: After the last assessment (12 months) participants are informed they have completed the study and receive no further contact from the research team.

Withdrawing involves no penalty or loss of benefits to which the participants are otherwise entitled. If the participants decide to quit the study all they have to do is tell the person in charge. It will not harm their relationship with their school or the researchers. They will receive no further contact from the research team after withdrawing.

6.3.7 Removal of Subjects

Participants can voluntarily withdraw from the study at any time. Additionally, the research staff may remove subjects from the study due to purposeful non-compliance with the study and school protocol and safety guidelines. If the subject is removed by study staff, they will be notified verbally and through writing. Parents/guardians of participants aged 16-17 will be notified as well.

6.4 Statistical Method

6.4.1 Statistical Design

For the statistical analyses, to assess the adequacy of randomization, participant baseline characteristics among the two groups will be evaluated using descriptive statistics (mean, median, IQR) and graphs. Baseline characteristics that are determined not to be equally distributed among the two groups will be considered for covariate adjustment to determine their impact on intervention comparisons. The primary hypothesis is that, at 3 months, there will be a higher proportion of PlaySmart vs. control participants who report greater risk of harm from misuse of prescription opioids AND heroin/fentanyl.

The primary comparison will be the main effects of PlaySmart vs. control games. This comparison will be made by comparing the proportion of participants in the two study arms who achieved the primary outcome. The probability of achieving the primary outcome will be

modeled using logistic regression. An unadjusted model (intervention variable only; stratified and non-stratified) and adjusted models (intervention plus other predictor variables) will be used in the analytical approach. In addition to 3 months, chi-square tests will be used to assess differences in proportions with the primary outcome in the two arms at 6 weeks, 6 and 12 months.

Differences in (a) intentions to misuse opioids and (b) self-efficacy for refusing opioids will be compared between the two groups. Means (standard errors) of intention and self-efficacy scores, by group, will be plotted at the various study time points (6 weeks, 3, 6 and 12 months). Change from baseline at each timepoint will also be compared between the two groups. The effective sample size of 532 provides >95% power (two-sided alpha-level 0.05; repeated measures ANOVA; G-Power v3.1.9.2) to detect an effect size of 0.15 (intervention group assumed to have a lower intention score) when comparing intentions to misuse opioids and self-efficacy for refusing opioids between the two study groups. Mixed Models (MM) will be used for longitudinal analysis to assess differences between the two groups over time in terms of (a) intention and (b) self-efficacy scores. MM is useful for unbalanced repeated measures with missing data, allows for intra-subject serial correlation and unequal variance and covariance structure across time, provides tests of the overall between-subject effects, repeated measures (time) effects, tests of fixed and random effects, and also allows analysis of reduced models that can provide detailed tests of specific pattern of results. The MM procedure will include a fixed effect for intervention group, time as a continuous variable (or categorical as appropriate), and the interaction between intervention and time to assess difference in slopes by intervention. Random effects will be included to account for intra-participant correlation of repeated measures. Model fit will be assessed with analysis of residuals and other methods of goodness-of-fit.

While the ultimate goal of this work is to prevent the initiation of opioid misuse in youth, it is not likely that a substantial proportion of even these high-risk adolescents will initiate opioid misuse during the 12-month follow-up period. Therefore, consistent with RFA-DA-19-035, our primary outcome is a "proximal target or specified mechanism presumed to underlie the intervention's effect on a distal outcome." We will compare the proportions of participants initiating opioid misuse at all follow-up timepoints (with a focus on 12 months) using chi-square. Given the observed rate of initiating opioid misuse in high school populations is 14%, and we are focusing on high-risk adolescents who report current substance use and symptoms of depression or anxiety, we conservatively estimate a three-fold increased likelihood of initiating opioid misuse resulting in 42% of the population initiating opioid misuse. Given the relationship between perceptions of harm and initiation of misuse, and a 15% reduction in initiation of opioid misuse based on exposure to the videogame intervention, we expect an absolute decrease in initiation of 6% (36% in the PlaySmart arm vs. 42% in the control arm) at 12 months. The effective sample size of 532 will provide 29% power (two-sided alpha-level 0.05) to detect this difference. The probability of achieving this outcome will also be modeled using logistic regression analysis. Stratified (using

randomization stratification variables; gender and grade) analysis and non-stratified analyses and adjusted models (intervention plus other predictor variables, mediator and moderator variables, e.g.) will be used in further logistic regression analyses.

Data will be collected on potential mediators and moderators. Because these variables might change over time during participation in the study, they will also be considered as time-varying covariates. Mediators: Based on our logic model and the literature that will guide the development of the proposed intervention, we predict that 1) knowledge, 2) perceived norms, and 3) attitudes will mediate the effects of the intervention on perception of risk of harm from misuse of opioids at 3 months. We will test the relationships among the outcomes and presumed mediators as proposed by MacKinnon et al. To test the significance of the mediation effect, which is called the indirect effect, we will calculate Sobel's test for each proposed mediator. Sobel's test determines the significance of the mediation effect by testing the null hypothesis that the indirect effect coefficient is zero (i.e., whether the indirect effect of the independent variable on the dependent variables is significantly different from zero). Moderators: Variables that predict the outcome variable (perceived risk of harm) differently between interventions will be considered to be moderators using the model outlined by Kraemer et al. To be considered a moderator, the variable must be present prior to randomization and must not be related to the independent variable (PlaySmart vs. control games). Decision-making skills as a moderator, will be compared between the two study arms at (a) the various time points; (b) change from baseline at various time points and overall study duration; (c) improvement (No/Yes change) and (d) longitudinally. We will use t-tests for continuous variables, chi-square for comparing proportion of participants who showed improvement from baseline to each study time point and overall, and longitudinal modeling to include measures of moderators over the whole study duration.

In the PlaySmart group, we plan to evaluate the prognostic significance of a small set of predictor variables (race/ethnicity, socioeconomic status) and plan to use them as covariate terms in the analyses of other study outcomes where appropriate. Alternatively, subgroups of participants characterized by the predictor variables can be compared on the selected outcome measures to better inform the interpretation of the significant study effects (i.e., to explore whether one type of intervention is most effective within particular subgroups).

While Dr. Fiellin will be notified of any adverse events to ensure the safety of the procedures, she will not be involved with any participant recruitment and/or data management nor data analysis. Tyra Pendergrass Boomer is appointed as the co-investigator who will be independent of Dr. Fiellin's review over subject selection and adverse event reporting responsibilities.

6.4.2 Sample Size Considerations

The sample size calculation, executed in PASS 2008, for this trial is based on the comparison of proportions of participants reporting great risk of harm from misuse of opioids at 3 months (primary outcome). The following assumptions were used: the approximated baseline rate of perception of great harm of risk from opioid misuse (composite outcome of endorsing great risk of harm from misuse of prescription opioids AND heroin/fentanyl) in high school students is 32%; randomization takes place over 2 years; participants are followed for 12 months; tests are two-sided done at the 0.05 level; two study arms (vs. control). A conservative estimate from the literature on drug use prevention interventions, and using data from a study examining the impact of an intervention on perceptions of harm of drug use, indicates that a sample size of 454 participants will be required to detect a 15% absolute difference in the proportion of participants achieving the primary outcome (reporting great risk of harm from misuse of prescription opioids and heroin/fentanyl) [50% (PlaySmart) vs. 35% (control)] with a power of 90%. To account for a 16% loss at 3 months based on our PlayForward study, the sample size is inflated to 532 participants (266 in each arm).

6.4.3 Planned Analyses

The primary comparison will be the main effects of PlaySmart vs. control games. This comparison will be made by comparing the proportion of participants in the two study arms who achieved the primary outcome. The probability of achieving the primary outcome will be modeled using logistic regression. An unadjusted model (intervention variable only; stratified and non-stratified) and adjusted models (intervention plus other predictor variables) will be used in the analytical approach. In addition to 3 months, chi-square tests will be used to assess differences in proportions with the primary outcome in the two arms at 6 weeks, 6 and 12 months.

Differences in (a) intentions to misuse opioids and (b) self-efficacy for refusing opioids will be compared between the two groups. Means (standard errors) of intention and self-efficacy scores, by group, will be plotted at the various study time points (6 weeks, 3, 6 and 12 months). Change from baseline at each timepoint will also be compared between the two groups. The effective sample size of 532 provides >95% power (two-sided alpha-level 0.05; repeated measures ANOVA; G-Power v3.1.9.2) to detect an effect size of 0.15 (intervention group assumed to have a lower intention score) when comparing intentions to misuse opioids and self-efficacy for refusing opioids between the two study groups. Mixed Models (MM) will be used for longitudinal analysis to assess differences between the two groups over time in terms of (a) intention and (b) self-efficacy scores. MM is useful for unbalanced repeated measures with missing data, allows for intra-subject serial correlation and unequal variance and covariance structure across time, provides tests of the overall between-subject effects, repeated measures (time) effects, tests of fixed and random effects, and also allows analysis of reduced models that can provide detailed tests of specific pattern of results. The MM procedure will include a fixed effect for intervention group, time as a continuous variable (or categorical as appropriate), and the interaction between intervention and time to assess difference in slopes by intervention. Random effects will be included to account for intra-

participant correlation of repeated measures. Model fit will be assessed with analysis of residuals and other methods of goodness-of-fit.

6.4.3.1 Primary Analyses

The primary comparison will be the main effects of PlaySmart vs. control games. This comparison will be made by comparing the proportion of participants in the two study arms who achieved the primary outcome. The probability of achieving the primary outcome will be modeled using logistic regression. An unadjusted model (intervention variable only; stratified and non-stratified) and adjusted models (intervention plus other predictor variables) will be used in the analytical approach. In addition to 3 months, chi-square tests will be used to assess differences in proportions with the primary outcome in the two arms at 6 weeks, 6 and 12 months.

6.4.3.2 Secondary Objectives Analyses, if applicable

Differences in (a) intentions to misuse opioids and (b) self-efficacy for refusing opioids will be compared between the two groups. Means (standard errors) of intention and self-efficacy scores, by group, will be plotted at the various study time points (6 weeks, 3, 6 and 12 months). Change from baseline at each timepoint will also be compared between the two groups. The effective sample size of 532 provides >95% power (two-sided alpha-level 0.05; repeated measures ANOVA; G-Power v3.1.9.2) to detect an effect size of 0.15 (intervention group assumed to have a lower intention score) when comparing intentions to misuse opioids and self-efficacy for refusing opioids between the two study groups. Mixed Models (MM) will be used for longitudinal analysis to assess differences between the two groups over time in terms of (a) intention and (b) self-efficacy scores. MM is useful for unbalanced repeated measures with missing data, allows for intra-subject serial correlation and unequal variance and covariance structure across time, provides tests of the overall between-subject effects, repeated measures (time) effects, tests of fixed and random effects, and also allows analysis of reduced models that can provide detailed tests of specific pattern of results. The MM procedure will include a fixed effect for intervention group, time as a continuous variable (or categorical as appropriate), and the interaction between intervention and time to assess difference in slopes by intervention. Random effects will be included to account for intra-participant correlation of repeated measures. Model fit will be assessed with analysis of residuals and other methods of goodness-of-fit.

6.4.3.3 Analysis of Subject Characteristics

N/A

6.4.3.4 Interim Analysis (if applicable)

We will establish a **Data Safety and Monitoring Board (DSMB)** comprised of two experts in clinical trials in adolescents (one with expertise in substance use prevention) and an expert in statistical analysis of clinical trials who will meet twice yearly to review study progress.The DSMB will monitor the occurrence and frequency of serious adverse events.

Given the relatively small number of participants that will be enrolled in the proposed study, the potential problems introduced with interim analyses, the low-risk nature of the intervention, and given we believe it is critical to evaluate for the durability of the

effect of the intervention at the follow-up timepoints of 6 and 12 months, we will not conduct an interim analysis.

6.4.3.5 Health economic evaluation, if applicable

6.4.3.6 Other

6.4.4 Subsets and Covariates

For the statistical analyses, to assess the adequacy of randomization, participant baseline characteristics among the two groups will be evaluated using descriptive statistics (mean, median, IQR) and graphs. Baseline characteristics that are determined not to be equally distributed among the two groups will be considered for covariate adjustment to determine their impact on intervention comparisons.

Mixed Models (MM) will be used for longitudinal analysis to assess differences between the two groups over time in terms of (a) intention and (b) self-efficacy scores. MM is useful for unbalanced repeated measures with missing data, allows for intra-subject serial correlation and unequal variance and covariance structure across time, provides tests of the overall between-subject effects, repeated measures (time) effects, tests of fixed and random effects, and also allows analysis of reduced models that can provide detailed tests of specific pattern of results. The MM procedure will include a fixed effect for intervention group, time as a continuous variable (or categorical as appropriate), and the interaction between intervention and time to assess difference in slopes by intervention.

Data will be collected on potential mediators and moderators. Because these variables might change over time during participation in the study, they will also be considered as time-varying covariates.

In the PlaySmart group, we plan to evaluate the prognostic significance of a small set of predictor variables (race/ethnicity, socioeconomic status) and plan to use them as covariate terms in the analyses of other study outcomes where appropriate. Alternatively, subgroups of participants characterized by the predictor variables can be compared on the selected outcome measures to better inform the interpretation of the significant study effects (i.e., to explore whether one type of intervention is most effective within particular subgroups).

6.4.5 Handling of Missing Data

Data questions or problems will trigger data queries and analyses of missing data will be done periodically to assure that all forms are entered and available for analysis. CTMS staff have received HIPAA training and Human Subjects Protection training. Users will certify that they are HIPAA trained and will act in full compliance of HIPAA regulations. Specifically, individual profile data and data on application use by study participants will be collected and de-identified through irreversible hashing methods prior to storage to ensure privacy protection is satisfied. De-identified data will be encrypted in transit and securely transferred for storage and analyses.

7.1 Ethical Considerations: Informed Consent/Assent and HIPAA Authorization

As part of participation in the RCT, adolescent participants will be provided compensation for completion of the baseline assessments ($55 gift card), assessments at 6 weeks immediately following completion of gameplay ($35), at 3, 6, and 12 months ($45 gift cards for each set of assessments). Additionally, students that randomize to experimental will also be given the opportunity to earn an additional $10 gift card to participate in qualitative interviews about their gameplay experience. The total possible compensation for the study is $225-$235 per adolescent participant.

Adult school-based health center staff will receive compensation for their participation in the randomized controlled trial and answering implementation surveys after the completion of the study. Health center staff would receive an honorarium of $5000 the first year of the study, $5000 the second year of the study, and $5000 the third year of the study.

One of the potential risks associated with this study pertains to the maintenance of the confidentiality of the identities of the participants enrolled in the study and information relating to them. De-identified hard copies of the data will be stored in locked cabinets and electronic data will be stored in secure, password-protected computers. Only the PI and other relevant research staff will have access to the data.

A number of precautions will be actively integrated into the research procedures to protect the confidentiality and anonymity of all participants. As we have done with our other videogame intervention studies, a Certificate of Confidentiality will be automatically provided by the National Institute on Drug Abuse upon their granting the award. All research staff participating in the study will be required to complete training in research ethics. Data collection forms will be designated by ID numbers only. A separate master file of names, addresses, contact persons, and telephone numbers, along with the study ID numbers will be maintained in a locked file cabinet in the PI's research offices. All data entry and analyses will be completed with ID numbers only. The study will be explained to others, such as the parent/guardian and program staff as a study of child development that will focus on promoting healthy behaviors. In instances in which data are requested from other sources or it is beneficial to the participant to provide information to another individual or agency (e.g. medical personnel) this will only be done with the written permission of the parent/guardian on a "Release of Information" form stipulating who the information is provided to, or received from. The research staff will follow standard confidentiality procedures for research programs.

Any breaches of confidentiality will be reported immediately to the participant, their family, and relevant school personnel as well as the IRB and the NIDA PO as well as the HIPAA Privacy officer. Additional systems will then be instituted to prevent a similar breach from occurring.

A member of the research team (in collaboration with the designated school liaison/champion) will screen for eligibility, discuss the study with the participant and their parent/guardian, and obtain written informed assent/electronic informed assent from the individual and written informed consent/ electronic informed consent from their parent/guardian (if under age 18). Participants will be provided with an age-appropriate description of the study as "finding out how teens can make choices that are healthier for them." In describing the study to the parent/guardian, we will indicate that the study is about promoting healthy behaviors and on providing strategies to assist adolescents in making decisions about the challenges they face, including the risk for initiating drug use. If an individual or their parent/guardian indicates that they do not wish to participate, there will be no further involvement in the study. In the case of those participants who are over the age of 18 (18-19 years old) or are emancipated minors, we will obtain written informed consent/ electronic informed consent from them.

Given the proposed study includes adolescent youth, we will be required in situations such as this to report any concerns that arise at the school as part of required mandatory reporting (all members of the research team have completed mandatory reporter training). This includes mandatory reporting for abuse and neglect to appropriate officials via Careline at 1-800-842-2288. Suicidal risk will be reported to school official as well as parent/guardian. In extreme cases, 9-1-1 will be called.

Fortunately, we will be working closely with the personnel of the school-based health center who are well-trained in managing these types of situations. We will employ all necessary measures for protecting the confidentiality of our participants who agree to be in this pilot study.

This research does not involve subjects with limited decision-making capacity. Parents and adolescents will be provided with an age-appropriate description of the study and told about what they might expect. In describing the study to the participant, the purpose of the study will be described as "finding out how kids can make choices that are healthier for them". In describing the study to the guardian/parent, we will indicate that the study will focus on promoting healthy behaviors in adolescence and on providing strategies to assist adolescents in making decisions about the many challenges they face, including risk for initiating drug use. Parents who do not wish for their children to participate may contact the project director. We will obtain written informed assent/electronic informed assent from the participant and written informed consent/ electronic informed consent from their guardian/parent.

7.2 Institutional Review Board (IRB) Review

This study presents minimal risks to the participants and adverse events or other problems are not anticipated.

Adverse Events (AEs):

Violation of confidentiality: Any breach of confidentiality.

Discomfort due to assessment procedures: Discomfort created by answering survey questions.

Embarrassment in disclosing sensitive personal information.

Disclosure of information about current and/or intended physical harm to persons; current and/or intended abuse of children that would be reported to a child welfare agency; and/or an investigation of such allegations(s) that could ensue.

Dissatisfaction with the intervention activities: In this case it would be the game intervention.

Serious Adverse Events (SAEs):

SAEs include death, life threatening injury or condition, hospitalization, persistent or significant disability/incapacity, and other conditions which in the judgment of the investigators represent significant hazards.

Procedures for research team management of AEs, SAEs and other study risks such as mandatory reporting requirements:

This study presents minimal risks to the participants and adverse events or other problems are not anticipated. In the unlikely event that such events occur, Reportable Adverse Events (which are events that are serious or life-threatening and unanticipated (or anticipated but occurring with a greater frequency than expected) and possibly, probably, or definitely related) or unanticipated problems involving risks to subjects or others will be reported in writing within 24 hours to the IRB (using the appropriate forms from the website) and the NIDA PO with a follow-up report within 72 hours. The PI will apprise fellow investigators and study personnel of all adverse events that occur during the conduct of this research project through regular study meetings and via email as they are reviewed by the PI. We will use procedures to detect and respond to adverse events that ensure prompt discovery of any adverse events and to minimize their effects. There is adequate surveillance and protections to discover adverse events promptly and keep their effects minimal.

Procedures for training and supervision of all research personnel have been developed as part of our previous and current studies. All members of the research team are familiar with procedures for identifying and reporting possible adverse reactions. All members of the research team are trained in mandatory reporting and will notify the PI immediately of any concerns raised about participant mental health (suicidality), substance use, abuse of any kind, and so forth.

If an adverse event occurs study personnel will notify the PI as well as the appropriate Information Security Officer (ISO) and follow all necessary procedures. All adverse events are reported using the Yale Institutional Review Board (IRB) standard template for reporting adverse events. The PI reviews all adverse events, classifies the attribution of adverse

events (e.g., definitely, probably, possibly related; unlikely or unrelated) and grades the severity of the event, utilizing the FDA's definition of serious adverse events, on a 6-point scale (0=no adverse event or within normal limit; 1=mild; 2=moderate; 3=severe; 4=life-threatening; 5=fatal). Serious unanticipated or anticipated adverse events will be reported immediately to the IRB and to the NIDA PO within 24 hours by phone and/or email and will submit a written report to the PO no more than two days later. Adverse events will be reported in summary form at least annually to the IRB. The summary will include the number of participants enrolled and a summary of graded adverse events to date, using the chart format included in the Yale University DSMP template. The PI will evaluate all adverse events and determine whether the event affects the Risk/Benefit ratio of the study and whether modifications to the protocol (e.g., Risks to Subjects) or consent form (e.g., Risks and Inconveniences) are required.

The PI will be responsible for monitoring the data and conducting performance and safety reviews, at the specified frequency. Either the PI or the IRB have the authority to stop or modify the study. The monitoring by the IRB will occur annually at the time of re-approval. The PI will conduct data and safety review at least quarterly and at any time a serious adverse event occurs. During the review process, the PI will evaluate whether the study should continue unchanged, requires modification or amendment to continue, or should be closed to enrollment.

7.3 Subject Privacy, Confidentiality & Data Management

A web-based computer system (RedCap) will be used for data collection, management, and monitoring. The web-based data system Clinical Trial Management System (CTMS) is under the auspices of the Yale Center for Medical Informatics who is responsible for data management. It is a web-accessible, multi-disciplinary database for study- or disease-specific clinical research data designed to store the focused data required for clinical trials and clinical research studies. This system has proven to be very efficient and reliable in other clinical trials. It will be used by the research assistant to administer the research instruments from any computer with Internet access. The CTMS will be used to generate a number of web accessible reports and reminders to help monitor and manage the data collection process to assure completeness of evaluation. The system can check for data inconsistencies, omissions, and errors regularly. Data questions or problems will trigger data queries and analyses of missing data will be done periodically to assure that all forms are entered and available for analysis. CTMS staff have received HIPAA training and Human Subjects Protection training. Users will certify that they are HIPAA trained and will act in full compliance of HIPAA regulations. Specifically, individual profile data and data on application use by study participants will be collected and de-identified through irreversible hashing methods prior to storage to ensure privacy protection is satisfied. De-identified data will be encrypted in transit and securely transferred for storage and analyses.

The web data-entry interface allows data entry to be performed from anywhere on the Internet and uses 128-bit secure sockets layer security to protect the confidentiality of the data. The CTMS maintains an electronic audit trail of all modifications to a study's data, including the user who made the change, date and time, each data item changed and its

previous value and new value. Yale houses and maintains the security and backup of all servers and workstations. The CTMS's Oracle database is housed in a central machine room maintained by Yale. Passwords and subject identifying data will be stored encrypted in the database server. Several levels of database backup are performed regularly, including full daily and incremental backup.

7.4 Deviations/Unanticipated Problems

Any protocol deviations or unanticipated problems will immediately be reported to the PI and IRB.

If an adverse event occurs study personnel will notify the PI as well as the appropriate Information Security Officer (ISO) and follow all necessary procedures. All adverse events are reported using the Yale Institutional Review Board (IRB) standard template for reporting adverse events. The PI reviews all adverse events, classifies the attribution of adverse events (e.g., definitely, probably, possibly related; unlikely or unrelated) and grades the severity of the event, utilizing the FDA's definition of serious adverse events, on a 6-point scale (0=no adverse event or within normal limit; 1=mild; 2=moderate; 3=severe; 4=life-threatening; 5=fatal). Serious unanticipated or anticipated adverse events will be reported immediately to the IRB and to the NIDA PO within 24 hours by phone and/or email and will submit a written report to the PO no more than two days later. Adverse events will be reported in summary form at least annually to the IRB. The summary will include the number of participants enrolled and a summary of graded adverse events to date, using the chart format included in the Yale University DSMP template. The PI will evaluate all adverse events and determine whether the event affects the Risk/Benefit ratio of the study and whether modifications to the protocol (e.g., Risks to Subjects) or consent form (e.g., Risks and Inconveniences) are required.

7.5 Data Collection

A web-based computer system (RedCap) will be used for data collection, management, and monitoring. The web-based data system Clinical Trial Management System (CTMS) is under the auspices of the Yale Center for Medical Informatics who is responsible for data management. It is a web-accessible, multi-disciplinary database for study- or disease-specific clinical research data designed to store the focused data required for clinical trials and clinical research studies. This system has proven to be very efficient and reliable in other clinical trials. It will be used by the research assistant to administer the research instruments from any computer with Internet access. The CTMS will be used to generate a number of web accessible reports and reminders to help monitor and manage the data collection process to assure completeness of evaluation. The system can check for data inconsistencies, omissions, and errors regularly. Data questions or problems will trigger data queries and analyses of missing data will be done periodically to assure that all forms are entered and available for analysis. CTMS staff have received HIPAA training and Human Subjects Protection training. Users will certify that they are HIPAA trained and will act in full compliance of HIPAA regulations. Specifically, individual profile data and data on application use by study participants will be collected and de-identified through irreversible hashing

methods prior to storage to ensure privacy protection is satisfied. De-identified data will be encrypted in transit and securely transferred for storage and analyses.

The web data-entry interface allows data entry to be performed from anywhere on the Internet and uses 128-bit secure sockets layer security to protect the confidentiality of the data. The CTMS maintains an electronic audit trail of all modifications to a study's data, including the user who made the change, date and time, each data item changed and its previous value and new value. Yale houses and maintains the security and backup of all servers and workstations. The CTMS's Oracle database is housed in a central machine room maintained by Yale. Passwords and subject identifying data will be stored encrypted in the database server. Several levels of database backup are performed regularly, including full daily and incremental backup.

All data is entered by the research assistant or by the participant with research assistant supervision if needed. All data is entered directly into the web-based computer system, RedCap. All data entry will be completed with assigned ID numbers only. The research team regularly and systematically double- checks and confirms the completeness of data entry.

7.6 Data Quality Assurance

A web-based computer system (RedCap) will be used for data collection, management, and monitoring. The web-based data system Clinical Trial Management System (CTMS) is under the auspices of the Yale Center for Medical Informatics who is responsible for data management. It is a web-accessible, multi-disciplinary database for study- or disease-specific clinical research data designed to store the focused data required for clinical trials and clinical research studies. This system has proven to be very efficient and reliable in other clinical trials. It will be used by the research assistant to administer the research instruments from any computer with Internet access. The CTMS will be used to generate a number of web accessible reports and reminders to help monitor and manage the data collection process to assure completeness of evaluation. The system can check for data inconsistencies, omissions, and errors regularly. Data questions or problems will trigger data queries and analyses of missing data will be done periodically to assure that all forms are entered and available for analysis. CTMS staff have received HIPAA training and Human Subjects Protection training. Users will certify that they are HIPAA trained and will act in full compliance of HIPAA regulations. Specifically, individual profile data and data on application use by study participants will be collected and de-identified through irreversible hashing methods prior to storage to ensure privacy protection is satisfied. De-identified data will be encrypted in transit and securely transferred for storage and analyses.

The web data-entry interface allows data entry to be performed from anywhere on the Internet and uses 128-bit secure sockets layer security to protect the confidentiality of the data. The CTMS maintains an electronic audit trail of all modifications to a study's data, including the user who made the change, date and time, each data item changed and its previous value and new value. Yale houses and maintains the security and backup of all servers and workstations. The CTMS's Oracle database is housed in a central machine

room maintained by Yale. Passwords and subject identifying data will be stored encrypted in the database server. Several levels of database backup are performed regularly, including full daily and incremental backup.

7.7 Study Records

Protocol

Consent

Adolescent Assent

SBHC Staff Information Sheet

Assessment surveys

7.8 Access to Source

A web-based computer system (RedCap) will be used for data collection, management, and monitoring. The web-based data system Clinical Trial Management System (CTMS) is under the auspices of the Yale Center for Medical Informatics who is responsible for data management. It is a web-accessible, multi-disciplinary database for study- or disease-specific clinical research data designed to store the focused data required for clinical trials and clinical research studies. This system has proven to be very efficient and reliable in other clinical trials. It will be used by the research assistant to administer the research instruments from any computer with Internet access. The CTMS will be used to generate a number of web accessible reports and reminders to help monitor and manage the data collection process to assure completeness of evaluation. The system can check for data inconsistencies, omissions, and errors regularly. Data questions or problems will trigger data queries and analyses of missing data will be done periodically to assure that all forms are entered and available for analysis. CTMS staff have received HIPAA training and Human Subjects Protection training. Users will certify that they are HIPAA trained and will act in full compliance of HIPAA regulations. Specifically, individual profile data and data on application use by study participants will be collected and de-identified through irreversible hashing methods prior to storage to ensure privacy protection is satisfied. De-identified data will be encrypted in transit and securely transferred for storage and analyses.

The web data-entry interface allows data entry to be performed from anywhere on the Internet and uses 128-bit secure sockets layer security to protect the confidentiality of the data. The CTMS maintains an electronic audit trail of all modifications to a study's data, including the user who made the change, date and time, each data item changed and its previous value and new value. Yale houses and maintains the security and backup of all servers and workstations. The CTMS's Oracle database is housed in a central machine room maintained by Yale. Passwords and subject identifying data will be stored encrypted in the database server. Several levels of database backup are performed regularly, including full daily and incremental backup.

7.9 Data or Specimen Storage/Security

The web data-entry interface allows data entry to be performed from anywhere on the Internet and uses 128-bit secure sockets layer security to protect the confidentiality of the data. The CTMS maintains an electronic audit trail of all modifications to a study's data, including the user who made the change, date and time, each data item changed and its previous value and new value. Yale houses and maintains the security and backup of all servers and workstations. The CTMS's Oracle database is housed in a central machine room maintained by Yale. Passwords and subject identifying data will be stored encrypted in the database server. Several levels of database backup are performed regularly, including full daily and incremental backup.

During each game play session, **Player Game State Data** and **Activity Logging Data** is automatically collected within the game and is exported and stored on the secured online server. Data will not be collected or stored locally on iPads and will only be accessible from the secured online server, which will be password protected and only accessible by the research staff.

De-identified hard copies of the data will be stored in locked cabinets and electronic data will be stored in secure, password-protected computers. Only the PI and other relevant research staff will have access to the data.

7.10 Retention of Records

Information that will be collected during the assessments and interviews will be erased 12 months after the completion of the study and after review of their content has been completed.

7.11 Study Monitoring

We will establish a **Data Safety and Monitoring Board (DSMB)** comprised of two experts in clinical trials in adolescents (one with expertise in substance use prevention) and an expert in statistical analysis of clinical trials who will meet twice yearly to review study progress and will also conduct an interim analysis. This will be conducted six months after 60 participants have been randomized to either of the treatment groups or when all of the 60 participants in each group have completed the treatment protocol, whichever comes first. The interim analysis will evaluate whether the proportion of participants engaged in the interactive prevention videogame group and the participants in the control group differ significantly in terms of the primary outcome (at $p<.001$ per Haybittle-Peto method).The DSMB will monitor the occurrence and frequency of serious adverse events and review the results of the interim analysis.

7.12 Data Safety Monitoring Plan

7.13 Study Modification

We will update the protocol any time a change has been made to the study and re-submit to IRB. Once approved by the IRB, changes will be implemented into the study.

7.14 Study Discontinuation

Although highly unlikely, the study will be discontinued if there are serious concerns pertaining to the efficacy, feasibility and most importantly, the safety of the subjects.

7.15 Study Completion

The study will complete on December 31st 2024. The IRB will be notified on this date.

7.16 Conflict of Interest Management Plan

We will include any possible conflict of interest statements on the consent and assent forms to notify participants.

7.17 Funding Source

This study is funded by the National Institutes of Health Helping to End Addiction Long-term Initiative.

7.18 Publication Plan

At the end of the RCT and data analysis, results will be submitted to a peer-reviewed journal and a community report will be shared with our partners.

Appendices

Appendix #	Title	Section	Topic
1	Outcome Measures	6 Methods	6.1.3 Selection of Instruments/Outcome Measures
2		6 Methods	6.2.1 Efficacy
3		6 Methods	6.3.1 Study Schedule

List of Tables

References

1. Centers for Disease Control and Prevention. Wide-ranging online data for epidemiologic research (WONDER). 2017; http://wonder.cdc.gov. Accessed December 30, 2018.
2. Gaither JR, Shabanova V, Leventhal JM. US national trends in pediatric deaths from prescription and illicit opioids, 1999-2016. JAMA Network Open. 2018;1(8):e186558. doi: 10.1001/jamanetworkopen.2018.6558.
3. Substance Abuse and Mental Health Services Administration. Reports and Detailed Tables From the 2017 National Survey on Drug Use and Health (NSDUH). 2018; https://www.samhsa.gov/data/sites/default/files/cbhsqreports/NSDUHDetailedTabs2017/NSDUHDetailedTabs2017.pdf. Accessed February 2, 2019.
4. Flynn AB, Falco M, Hocini S. Independent evaluation of middle school–based drug prevention curricula: A systematic review. JAMA Pediatrics. 2015;169(11):1046-1052. doi: 10.1001/jamapediatrics.2015.1736.
5. Anderson M, Jiang J. Teens, Social Media & Technology 2018; http://www.pewinternet.org/2018/05/31/teens-social-media-technology-2018/. Accessed January 19, 2019.
6. Hieftje K, Edelman EJ, Camenga DR, Fiellin LE. Electronic media-based health interventions for behavior change in youth: A systematic review. JAMA Pediatrics. 2013;1-7. doi: 10.1001/jamapediatrics.2013.1095.
7. Fiellin LE, Hieftje KD, Pendergrass TM, et al. A videogame intervention for sexual risk reduction in minority adolescents: A randomized controlled trial. Journal of Medical Internet Research. 2017;19(9):e314. doi: 10.2196/jmir.8148.
8. Pentz MA, Hieftje KD, Pendergrass TM, et al. A videogame intervention for tobacco product use prevention in adolescents. Addictive Behaviors. 2019;91:188-192. doi: 10.1016/j.addbeh.2018.11.016.
9. Fiellin LE, Kyriakides TC, Hieftje KD, et al. The design and implementation of a randomized controlled trial of a risk reduction and HIV prevention videogame intervention in minority adolescents: PlayForward: Elm City Stories. Clinical Trials: Journal of the Society for Clinical Trials. 2016:1-9 doi:10.1177/1740774516637871.
10. Peng W. Design and evaluation of a computer game to promote a healthy diet for young adults. Health Communication. 2009;24(2):115-127. doi: 10.1080/10410230802676490.
11. Kato PM, Cole SW, Bradlyn AS, Pollock BH. A video game improves behavioral outcomes in adolescents and young adults with cancer: A randomized trial. Pediatrics. 2008;122(2):e305-317. doi: 10.1542/peds.2007-3134.
12. Hieftje K, Pendergrass TM, Montanaro E, Kyriakides T, Florsheim O, Fiellin LE. But do they like it?" Participant satisfaction and gameplay experience of a videogame intervention in adolescents. 2018 IEEE 6th International Conference on Serious Games and Applications for Health (SeGAH); 2018; Vienna, Austria.
13. Hieftje KD, Duncan LR, Fiellin LE. Novel methods to collect meaningful data from adolescents for the development of health interventions. Health Promotion Practice. 2014: doi: 10.1177/1524839914521211.
14. Hieftje KD, Duncan LR, Pendergrass TM, Sawyer B, Fiellin LE. Development of a HIV prevention videogame: Lessons learned. International Journal of Serious Games. 2016;3(2):83-90.
15. Hieftje KD, Fernandes CSF, Lin IH, Fiellin LE. Impact of a web-based tobacco product use prevention videogame intervention on adolescents. Nicotine & Tobacco Research (Under Review).
16. Hieftje KD, Rosenthal MS, Camenga DR, Edelman EJ, Fiellin LE. A qualitative study to inform the development of a videogame for adolescent human immunodeficiency virus prevention. Games for Health Journal: Research, Development, and Clinical Applications. 2012;1(4):1-5. doi: 10.1089/g4h.2012.0025.
17. Fiellin LE, Hieftje KD, Duncan LR. Videogames, here for good. Pediatrics. 2014;134(5):849-851. doi:10.1542/peds.2014-0941.

18. Fiellin LE, Gay G, Thompson DI, Baranowski T. Using what's learned in the game for use in real life: Roundtable discussion. Games for Health Journal. 2014;3(1):6-9. doi: 10.1089/g4h.2014.1715.
19. Duncan LR, Hieftje KD, Culyba S, Fiellin LE. Game Playbooks: Tools to guide multidisciplinary teams in developing videogame-based behavior change interventions. Translational Behavioral Medicine. 2014; doi: 10.1007/s13142-013-0246-8.
20. Duncan LR, Hieftje KD, Fiellin LE. PlayForward: Elm City Stories: Rationale and curriculum content of a videogame intervention for HIV prevention among at-risk adolescents. Manuscript in preparation.
21. Duncan LR, Hieftje KD, Pendergrass TM, Sawyer BG, Fiellin LE. Preliminary investigation of a videogame prototype for cigarette and marijuana prevention in adolescents. Substance Abuse 39(3):275-279. doi: 10.1080/08897077.2018.1437862.
22. Montanaro E, Fiellin LE, Fakhouri T, Kyriakides T, Duncan LR. Using videogame applications to assess gains in health-related knowledge in adolescents: New opportunities for evaluating intervention exposure and content mastery. Journal of Medical Internet Research. 2015;17(10):e245 doi: 210.2196/jmir.4377.
23. Pendergrass TM, Hieftje K, Crusto C, Montanaro E, Fiellin LE. If we build it, will they come? A qualitative study of key stakeholder opinions on the implementation of a videogame intervention for risk reduction in adolescents. Games for Health Journal: Research, Development, and Clinical Applications 2016;5(4):1-7. doi: 10.1089/g4h.2015.0092.
24. Pendergrass TM, Hieftje KD, Duncan LR, Fiellin LE. A brief report: Intervention to encourage HIV testing and counseling among adolescents. American Journal of Health Promotion (Under Review).
25. Camenga DR, Fiellin LE, Pendergrass TM, Miller E, Pentz MA, Hieftje KD. Adolescents' perceptions of flavored tobacco products, including e-cigarettes: A qualitative study to inform FDA tobacco education efforts through videogames. Addictive Behaviors. 2018;82:189-194. doi: 10.1016/j.addbeh.2018.03.021.
26. Camenga DR, Hieftje KD, Fiellin LE, Edelman EJ, Rosenthal MS, Duncan LR. The use of message framing to promote sexual risk reduction in young adolescents: A pilot exploratory study. Health Education Research. 2014; doi: 10.1093/her/cyt156.
27. Ajzen I. The theory of planned behavior. Organizational Behaviorand Human Decision Processes. 1991;50(2):179-211.
28. Bandura A. Self-efficacy: Toward a unifying theory of behavioral change. Psychological Review. 1977;84:191-215.
29. Bandura A. Self-efficacy: The exercise of control. New York: Freeman; 1997.
30. Rothman AJ, Salovey P. Shaping perceptions to motivate healthy behaviors: The role of message framing. Psychological Bulletin. 1997;121:3-19.
31. Tversky A, Kahneman D. The framing of decisions and the psychology of choice. Science. 1981;211(4481):453-458.
32. National Institute of Drug Abuse. National Institute on Drug Abuse 2016-2020 Strategic Plan. 2015.
33. Camenga DR, Hieftje KD, Fiellin LE, Edelman EJ, Rosenthal MS, Duncan LR. The use of message framing to promote sexual risk reduction in young adolescents: a pilot exploratory study. Health education research. 2014. doi: 10.1093/her/cyt156.
34. Miech RA, Johnston LD, O'Malley PM, Bachman JG, Schulenberg JE, Patrick ME. Monitoring the Future national survey results on drug use: 1975-2017: Volume I, secondary school students: 2017 Overview, key findings on adolescent drug use. Ann Arbor 2018.
35. Curtin SC, Tejad-Vera B, Warner M. Drug overdose deaths among adolescents aged 15-19 in the United States: 1999-2015. NCHS data brief, no 282 2017.
36. Substance Abuse and Mental Health Services Administration Center for Behavioral Health Statistics and Quality. The NSDUH Report: Trends in exposure to substance use prevention messages among adolescents. Rockville, MD February 7, 2013 2013.
37. Miech RA, Johnston LD, O'Malley PM, Bachman JG, Schulenberg JE, Patrick ME. Monitoring the Future national survey results on drug use, 1975–2017: Volume I, Secondary school students. University of Michigan. 2018.

38. Bachman J, Johnston L, O'Malley P, Humphrey R. Explaining the recent decline in marijuana use: Differentiating the effects of perceived risks, disapproval, and general lifestyle factors. Journal of Health and Social Behavior. 1988;29:92-112.
39. Collins D, Abadi MH, Johnson K, Shamblen S, Thompson K. Non-medical use of prescription drugs among youth in an Appalachian population: Prevalence, predictors, and implications for prevention.Journal of Drug Education. 2011;41(3):309-326. doi: 10.2190/DE.41.3.e.
40. Arria AM, Caldeira KM, Vincent KB, O''Grady KE, Wish ED. Perceived harmfulness predicts nonmedical use of prescription drugs among college students: Interactions with sensation seeking. Prevention Science. 2008;9(3):191-201. doi: 10.1007/s11121-008-0095-8.
41. Ford JA, Rigg KK. Racial/ethnic differences in factors that place adolescents at risk for prescription opioid misuse. Prevention Science. 2015;16:633-641. doi: 10.1007/s11121-014-0514-y.
Page 149
42. Voepel-Lewis T, Boyd CJ, McCabe SE, et al. Deliberative prescription opioid misuse among adolescents and emerging adults: Opportunities for targeted interventions. Journal of Adolescent Health. 2018;63(5):594-600. doi: 10.1016/j.jadohealth.2018.07.007.
43. Votawa VR, Wittenauera J, Connery HS, Weissa RD, McHugh RK. Perceived risk of heroin use among nonmedical prescription opioid users. Addictive Behaviors. 2017;65:218-223. doi: 10.1016/j.addbeh.2016.08.025.
44. Chou C-P, Montgomery SB, Pentz MA, Rohrbach LA, Johnson CA, Flay BR, MacKinnon D. Effects of a community-based prevention program on decreasing drug use in high risk adolescents. American Journal of Public Health. 1998;88(6):944-948.
45. Fiellin LE, Tetrault JM, Becker WC, Fiellin DA, Hoff RA. Previous use of alcohol, cigarettes, and marijuana and subsequent abuse of prescription opioids in young adults. Journal of Adolescent Health. 2013;52(2):158-163. doi: 10.1016/j.jadohealth.2012.06.010.
46. Martins SS, Fenton MC, Keyes KM, Blanco C, Zhu H, Storr CL. Mood/anxiety disorders and their association with non-medical prescription opioid use and prescription opioid use disorder: Longitudinal evidence from the National Epidemiologic Study on Alcohol and Related Conditions. Psychological Medicine. 2012;42(6):1261-1272. doi: 10.1017/S0033291711002145.
47. Kirby D. School-based clinics: Research results and their implications for future research methods.Evaluation and Program Planning. 1991;14(1-2):35-47. doi: 10.1016/0149-7189(91)90038-I.
48. Kisker EE, Brown RS. Do school-based health centers improve adolescents' access to health care, health status, and risk-taking behavior? Journal of Adolescent Health. 1996;18(5):335-343. doi: 10.1016/1054-139X(95)00236-L.
49. Parasuraman SR, Shi L. The Role of School-Based Health Centers in Increasing Universal and Targeted Delivery of Primary and Preventive Care Among Adolescents. Journal of School Health. 2014;84(8):524-532. doi: 10.1111/josh.12178.
50. Walker SC, Kerns SE, Lyon AR, Bruns EJ, Cosgrove T. Impact of school-based health center use on academic outcomes. Journal of Adolescent Health. 2010;46(3):251-257. doi: 10.1016/j.jadohealth.2009.07.002.
51. Bersamin M, Paschall MJ, Fisher DA. School-Based Health Centers and Adolescent Substance Use: Moderating Effects of Race/Ethnicity and Socioeconomic Status. Journal of School Health. 2017;87(11):850-857. doi: 10.1111/josh.12559.
52. Klein JD, Handwerker L, Sesselberg TS, Sutter E, Flanagan E, Gawronski B. Measuring quality of adolescent preventive services of health plan enrollees and school-based health center users. Journal of Adolescent Health. 2007;41(2):153-160. doi: 10.1016/j.jadohealth.2007.03.012.
53. Salerno J, Darling-Fisher C, Hawkins NM, Fraker E. Identifying relationships between high-risk sexual behaviors and screening positive for chlamydia and gonorrhea in school-wide screening events. Journal of School Health. 2013;83(2):99-104. doi: 10.1111/josh.12004.
54. Shaffer DW, Squire KR, Halverson R, Gee JP. Video games and the future of learning. Phi Delta Kappan 2005;87(2):105-111.
55. Lave J, Wenger E. Situated learning: Legitimate peripheral participation. New York, NY: Cambridge University Press; US; 1991.

56. Lieberman DA. Interactive video games for health promotion: Effects on knowledge, self-efficacy, social support, and health. Health promotion and interactive technology: Theoretical applications and future
directions. Mahwah, NJ: Lawrence Erlbaum Associates Publishers; US; 1997:103-120.
57. Street RL, Jr., Rimal RN. Health promotion and interactive technology: A conceptual foundation. Health promotion and interactive technology: Theoretical applications and future directions. Mahwah, NJ: Lawrence Erlbaum Associates Publishers; US; 1997:1-18.
58. Ferguson W. The emergence of games for health. Games for Health Journal. 2012;1(2):1-2. doi: 10.1089/g4h.2012.1010.
59. Tarini P. Curiosity, hope, and vision: The role of the Robert Wood Johnson Foundation's Pioneer Portfolio in funding early health game research. Games for Health Journal. 2012;1(1):7-8. doi: 10.1089/g4h.2012.1018.
60. Merry SN, Stasiak K, Shepherd M, Frampton C, Fleming T, Lucassen MFG. The effectiveness of SPARX, a computerised self help intervention for adolescents seeking help for depression: randomized controlled non-inferiority trial. BMJ. 2012;344:e2598. doi: 10.1136/bmj.e2598.
61. Lieberman DA. Management of chronic pediatric diseases with interactive health games: Theory and research findings. The Journal of Ambulatory Care Management. 2001;24(1):26-38.
Page 150
62. Krishna S, Francisco BD, Balas EA, et al. Internet-enabled interactive multimedia asthma education program: A randomized trial. Pediatrics. 2003;111(3):503-510.
63. Horan PP, Yarborough MC, Besigel G, Carlson DR. Computer-assisted self-control of diabetes by adolescents. The Diabetes Educator. 1990;16(3):205-211. doi: 10.1177/014572179001600311.
64. Brown SJ, Lieberman DA, Gemeny BA, Fan YC, Wilson DM, Pasta DJ. Educational video game for juvenile diabetes: Results of a controlled trial. Medical Informatics. 1997;22(1):77-89.
65. Bainbridge W. The scientific research potential of virtual worlds. Science. 2007;317:472-476. doi: 10.1126/science.1146930.
66. Schwebel DC, Gainesa J, Severson J. Validation of virtual reality as a tool to understand and prevent child pedestrian injury. Accident Analysis & Prevention. 2008;40(4):1394-1400. doi: 10.1016/j.aap.2008.03.005.
67. Hubal R, Kizakevich P, Furberg R. Synthetic characters in health-related applications. In: S. Vaidya LCJ, & H. Yoshida, ed. Advanced Computational Intelligence Paradigms in Healthcare 2. Heidelberg, Germany: Springer-Verlag; 2007.
68. Johnson WL, Beal C. Iterative evaluation of an intelligent game for language learning. Paper presented at: Proceedings of the International Conference on Artificial Intelligence in Education. 2005; Amsterdam.
69. Marsch LA, Bickel WK, Grabinski MJ. Application of interactive, computer technology to adolescent substance abuse prevention and treatment. Adolescent Medicine: State of the Art Reviews. 2007;18(2):342-356.
70. Ruggiero D, Mong C. The Teacher Technology Integration Experience: Practice and Reflection in the Classroom. Journal of Information Technology Education: Research. 2015;14:161-178.
71. Pentz MA. Evidence-based prevention: Characteristics, impact, and future direction. Journal of Psychoactive Drugs. 2003;35(Suppl 1):143-152. doi: 10.1080/02791072.2003.10400509.
72. Hansen WB. Prevention programs: Factors that individually focused programs must address. Rockville, MD: 1997.
73. Griffin KW, Botvin GJ. Evidence-based interventions for preventing substance use disorders in adolescents. Child and Adolescent Psychiatric Clinics of North America. 2010;19(3):505-526. doi: 10.1016/j.chc.2010.03.005.
74. Gates S, McCambridge J, Smith LA, Foxcroft D. Interventions for prevention of drug use by young people delivered in non-school settings. 2006.
75. Springer JF, Sale E, Hermann J, Sambrano S, Kasim R, Nistler M. Characteristics of effective substance abuse prevention programs for high-risk youth. Journal of Primary Prevention. 2004;25(2):171-194.
76. Robertson EB, David SL, Rao SA. Preventing drug use among children and adolescents: A researchbased

guide for parents, educators, and community leaders. Bethesda, MD: National Institute on Drug Abuse; 2003.
77. Spoth R, Trudeau L, Shin C, et al. Longitudinal effects of universal preventive intervention on prescription drug misuse: three randomized controlled trials with late adolescents and young adults. American Journal of Public Health. 2013;103:665-672. doi: 10.2105/AJPH.2012.301209.
78. Crowley DM, Jones DE, Coffman DL, Greenberg MT. Can we build an efficient response to the prescription drug abuse epidemic? Assessing the cost effectiveness of universal prevention in the PROSPER trial. Preventive Medicine. 2014;62:71-77. doi: 10.1016/j.ypmed.2014.01.029.
79. Pentz MA, Dwyer JH, MacKinnon DP, et al. A multi-community trial for primary prevention of adolescent
drug abuse: Effects on drug use prevalence. Journal of the American Medical Association. 1989;261(22):3259-3266.
80. Riggs NR, & Pentz MA. Long-term effects of adolescent marijuana use prevention on adult mental health services utilization: the midwestern prevention project. Substance Use & Misuse. 2009;44(5):616-631. doi: 10.1080/10826080902809691.
81. Rimer BK, Glanz K. Theory at a glance: A guide for health promotion practice (NIH Publication No. 05-3896). Washington, DC 2005.
82. Sussman S, Dent CW, Leu L. The one-year prospective prediction of substance abuse and dependence among high-risk adolescents. Journal of Substance Abuse. 2000;12(4):373-386.
83. Andrews JA, Tildesley E, Hops H, Duncan SC, Severson HH. Elementary school age children's future intentions and use of substances. Journal of Clinical Child & Adolescent Psychology. 2003;32(4):556-567. doi: 10.1207/S15374424JCCP3204_8.
84. Maddahian E, Newcomb MD, Bentler PM. Adolescent drug use and intention to use drugs: concurrent and longitudinal analyses of four ethnic groups. Addictive Behaviors. 1988;13(2):191-195.
85. Langlois MA, Petosa R, Hallam JS. Why do effective smoking prevention programs work? Student changes in social cognitive theory constructs. Journal of School Health. 1999;8:326-331.
86. Perry CL, Williams CL, Veblen-Mortenson S, et al. Project Northland: outcomes of a communitywide alcohol use prevention program during early adolescence. American Journal of Public Health. 1996;86(7):956-965.
87. Jones RT, McDonald DW, Fiore MF, Arrington T, Randall J. A primary preventive approach to children's drug refusal behavior: The impact of rehearsal-plus. Journal of Pediatric Psychology. 1990;15(2):211-223.
88. Stephens PC, Sloboda Z, Stephens RC, et al. Universal school-based substance abuse prevention programs: Modeling targeted mediators and outcomes for adolescent cigarette, alcohol and marijuana use. Drug & Alcohol Dependence. 2009;102(1-2):19-29. doi: 10.1016/j.drugalcdep.2008.12.016.
89. Riggs NR, Chou C, Pentz MA. Preventing growth in amphetamine use: long-term effects of the Midwestern Prevention Project (MPP) from early adolescence to early adulthood. Addiction. 2009;104(10):1691-1699. doi: 10.1111/j.1360-0443.2009.02666.x.
90. Riggs NR, Elfenbaum P, Pentz MA. Parent program component analysis in a drug abuse prevention trial. Journal of Adolescent Health. 2006;39(1):66-72. doi: 10.1016/j.jadohealth.2005.09.013.
91. Chou CP, Montgomery S, Pentz MA, Rohrbach LA, Johnson CA, Flay BR, MacKinnon DP. Effects of a community-based prevention program on decreasing drug use in high-risk adolescents. American Journal of Public Health. 1998;88(6):944-948.
92. Pentz MA, Dwyer JH, MacKinnon DP, et al. A multicommunity trial for primary prevention of adolescent drug abuse. Effects on drug use prevalence. JAMA. 1989;261(22):3259-3266.
93. Pentz MA, Mackinnon D, Flay BR, Hansen WB, Johnson CA, Dwyer JH. Primary prevention of chronic diseases in adolescence: effects of the Midwestern Prevention Project on tobacco use. American Journal of Epidemiology. 1989;130(4):713-724.
94. Kahneman D, Tversky A. Prospect theory: An analysis of decisions under risk. Econometrica. 1979;47:263-291.
95. Kahneman D, Tversky A. Choices, values, and frames. American Psychologist. 1984;39(4):341-350.
96. Schinke SP, Di Noia J, Glassman JR. Computer-mediated intervention to prevent drug abuse and

violence among high-risk youth. Addictive Behaviors. 2004;29(1):225-229.
97. Schwinn TM, Schinke SP. Preventing alcohol use among late adolescent urban youth: 6-year results from a computer-based intervention. Journal of Studies on Alcohol & Drugs. 2010;71(4):535-538.
98. Sullivan LE, Fiellin DA. Narrative review: buprenorphine for opioid-dependent patients in office practice. Annals of Internal Medicine. 2008;148(9):662-670.
99. Sullivan LE, Moore BA, Chawarski MC, et al. Buprenorphine/naloxone treatment in primary care is associated with decreased human immunodeficiency virus risk behaviors. Journal of Substance Abuse Treatment. 2008;35(1):87-92. doi: 10.1016/j.jsat.2007.08.004.
100. Sullivan LE, Moore BA, O'Connor PG, et al. The association between cocaine use and treatment outcomes in patients receiving office-based buprenorphine/naloxone for the treatment of opioid dependence. American Journal on Addictions. 2010;19(1):53-58. doi: 10.1111/j.1521-0391.2009.00003.x.
101. Tetrault JM, Desai RA, Becker WC, Fiellin DA, Concato J, Sullivan LE. Gender and non-medical use of prescription opioids: results from a national US survey. Addiction. 2008;103(2):258-268. doi: 10.1111/j.1360-0443.2007.02056.x.
102. Edelman EJ, Chantarat T, Caffrey S, et al. The impact of buprenorphine/naloxone treatment on HIV risk behaviors among HIV-infected, opioid-dependent patients. Drug & Alcohol Dependence. 2014;Mar 15; doi: 10.1016/j.drugalcdep.2014.03.006.
103. Edelman JE, Dinh AT, Moore BA, Schottenfeld RS, Fiellin DA, Sullivan LE. Human immunodeficiency virus testing practices among buprenorphine-prescribing physicians. Journal of Addiction Medicine. 2012;6(2):159-165. doi: 10.1097/ADM.0b013e31824339fc.
104. Edelman EJ, Moore BA, Caffrey S, et al. HIV testing and sexual risk reduction counseling in officebased buprenorphine. Journal of Addiction Medicine. 2013;7(6):410-416. doi: 10.1097/ADM.0b013e3182a3b603.
105. Becker WC, Sullivan LE, Tetrault JM, Desai RA, Fiellin DA. Non-medical use, abuse and dependence on prescription opioids among U.S. adults: Psychiatric, medical and substance use correlates. Drug & Alcohol Dependence. 2008;94(1-3):38-47. doi: 10.1016/j.drugalcdep.2007.09.018.
106. Sullivan LE, Barry D, Moore BA, et al. A trial of integrated buprenorphine/naloxone and HIV clinical care. Clinical Infectious Diseases. 2006;43 Suppl 4:S184-190. doi: 10.1086/508182.
107. Sullivan LE, Chawarski M, O'Connor PG, Schottenfeld RS, Fiellin DA. The practice of office-based buprenorphine treatment of opioid dependence: Is it associated with new patients entering into treatment? Drug & Alcohol Dependence. 2005;79:113-116. doi: 10.1016/j.drugalcdep.2004.12.008.
108. Edelman EJ, Moore BA, Holt SR, et al. Efficacy of extended-release naltrexone on HIV-related and drinking outcomes among HIV positive patients: A randomized controlled trial. AIDS and Behavior. 2019;23(1):211-221. doi: 10.1007/s10461-018-2241-z.
109. Sullivan LE, Botsko M, Cunningham CO, et al. The impact of cocaine use on outcomes in HIV-infected patients receiving buprenorphine/naloxone. Journal of Acquired Immune Deficiency Syndromes.
2011;56(Suppl 1):S54-61. doi: 10.1097/QAI.0b013e3182097576.
110. Fiellin DA, Weiss L, Botsko M, et al. Drug treatment outcomes among HIV-infected opioid-dependent patients receiving buprenorphine/naloxone. Journal of Acquired Immune Deficiency Syndromes. 2011;Suppl 1:S33-38. doi: 10.1097/QAI.0b013e3182097537.
111. Schackman BR, Leff JA, Botsko M, et al. The cost of integrated HIV care and buprenorphine/naloxone treatment: results of a cross-site evaluation. Journal of Acquired Immune Deficiency Syndromes. 2011;56 Suppl 1:S76-82. doi: 10.1097/QAI.0b013e31820a9a66.
112. Korthuis PT, Fiellin DA, Fu R, et al. Improving adherence to HIV quality of care indicators in persons with opioid dependence: the role of buprenorphine. Journal of Acquired Immune Deficiency Syndromes. 2011;56 Suppl 1:S83-90. doi: 10.1097/QAI.0b013e31820bc9a5.
113. Korthuis PT, Saha S, Chander G, et al. Substance use and the quality of patient-provider communication in HIV clinics. AIDS and Behavior. 2011;15(4):832-841. doi: 10.1007/s10461-010-9779-8.

114. Chaudhry AA, Botsko M, Weiss L, et al. Participant characteristics and HIV risk behaviors among individuals entering integrated buprenorphine/naloxone and HIV care. Journal of Acquired Immune Deficiency Syndromes. 2011;56 Suppl 1:S14-21. doi: 10.1097/QAI.0b013e318209d3b9.
115. Altice FL, Bruce RD, Lucas GM, et al. HIV treatment outcomes among HIV-infected, opioid-dependent patients receiving buprenorphine/naloxone treatment within HIV clinical care settings: results from a multisite study. Journal of Acquired Immune Deficiency Syndromes. 2011;56 Suppl 1:S22-32. doi: 10.1097/QAI.0b013e318209751e.
116. Lum PJ, Little S, Botsko M, et al. Opioid-prescribing practices and provider confidence recognizing opioid analgesic abuse in HIV primary care settings. Journal of Acquired Immune Deficiency Syndromes. 2011;56 Suppl 1:S91-97. doi: 10.1097/QAI.0b013e31820a9a82.
117. Vergara-Rodriguez P, Tozzi MJ, Botsko M, et al. Hepatic safety and lack of antiretroviral interactions with buprenorphine/naloxone in HIV-infected opioid-dependent patients. Journal of Acquired Immune Deficiency Syndromes. 2011;56 Suppl 1:S62-67. doi: 10.1097/QAI.0b013e31820a820f.
118. Weiss L, Egan JE, Botsko M, Netherland J, Fiellin DA, Finkelstein R. The BHIVES collaborative: organization and evaluation of a multisite demonstration of integrated buprenorphine/naloxone and HIV treatment. Journal of Acquired Immune Deficiency Syndromes. 2011;56 Suppl 1:S7-13. doi: 10.1097/QAI.0b013e3182097426.
119. Weiss L, Netherland J, Egan JE, et al. Integration of buprenorphine/naloxone treatment into HIV clinical care: lessons from the BHIVES collaborative. Journal of Acquired Immune Deficiency Syndromes. 2011;56 Suppl 1:S68-75. doi: 10.1097/QAI.0b013e31820a8226.
120. Fernandes CS, Schwartz MB, Basch CE. Educator perspectives: Selected barriers to implementation of school-level nutrition policies. Journal of Nutrition Education and Behavior (In Press). doi:10.1016/j.jneb.2018.12.011.
121. Fiellin DA, Barry DT, Sullivan LE, et al. A Randomized Trial of Cognitive Behavioral Therapy in Primary Care-based Buprenorphine. American Journal of Medicine. 2013;126(1):e11-17. doi: 10.1016/j.amjmed.2012.07.005.
122. Fiellin DA, Barthwell AG. Guideline development for office-based pharmacotherapies for opioid dependence. Journal of Addictive Diseases. 2003;22:109-120.
123. Fiellin DA, Kleber HD, Trumble-Hejduk JG, McLellan AT, Kosten TR. Consensus statement on officebased treatment of opioid dependence using buprenorphine. Journal of Substance Abuse Treatment. 2004;27:153-159. doi: 10.1016/j.jsat.2004.06.005.
124. Fiellin DA, O'Connor PG. New Federal initiatives to enhance the medical treatment of opioid dependence. Annals of Internal Medicine. 2002;137:688-692.
125. Fiellin DA, O'Connor PG. Office-based treatment of opioid dependent patients. New England Journal of Medicine. 2002;347(11):817-823. doi: 10.1056/NEJMcp013579.
126. Fiellin DA, O'Connor PG, Chawarski M, Pakes JP, Pantalon MV, Schottenfeld RS. Methadone maintenance in primary care: A randomized controlled trial. JAMA. 2001;286:1724-1731.
127. Fiellin DA, O'Connor PG, Chawarski M, Pantalon MV, Schottenfeld RS. Office versus narcotic treatment program-based buprenorphine for opioid dependence. Drug and Alcohol Dependence. 2002;66(Suppl 1):S55-56.
128. Fiellin DA, O'Connor PG, Chawarski M, Schottenfeld RS. Processes of care during a randomized trial of office-based treatment of opioid dependence in primary care. American Journal on Addictions. 2004;13:S67-78. doi: 10.1080/10550490490440843.
129. Fiellin DA, Pantalon MV, Chawarski MC, et al. Counseling plus buprenorphine-naloxone therapy for opioid dependence. New England Journal of Medicine. 2006;355(4):365-374. doi: 10.1056/NEJMoa055255.
130. Fiellin DA, Pantalon MV, Pakes JP, O'Connor PG, Chawarski MC, Schottenfeld RC. Treatment of heroin dependence with buprenorphine in primary care. The American Journal of Drug and Alcohol Abuse. 2002;28(2):231-241.
131. Fiellin DA, Rosenheck RA, Kosten TR. Office-based treatment for opioid dependence: reaching new patient populations. American Journal of Psychiatry. 2001;158(8):1200-1204. doi: 10.1176/appi.ajp.158.8.1200.
132. Fiellin DA, Schottenfeld RS, Cutter CJ, Moore BA, Barry DT, O'Connor PG. Primary care-based

buprenorphine taper vs maintenance therapy for prescription opioid dependence: a randomized clinical trial. JAMA Internal Medicine. 2014;174(12):1947-1954. doi: 10.1001/jamainternmed.2014.5302.
133. Edelman EJ, Hansen NB, Cutter CJ, et al. Implementation of integrated stepped care for unhealthy alcohol use in HIV clinics. Addiction Science & Clinical Practice. 2016;11(1):1. doi: 10.1186/s13722-
015-0048-z.134. Stephan SH, Connors EH, Arora P, Brey L. A learning collaborative approach to training school-based health providers in evidence-based mental health treatment. Children and Youth Services Review 2013;35:1970-1978. doi: 10.1016/j.childyouth.2013.09.008.
135. Stephan S, Mulloy M, Brey L. Improving collaborative mental health care by school-based primary care and mental health providers. School Mental Health. 2011;3:70-80.
136. Scudder L, Papa P, Brey LC. School-based health centers: A model for improving the health of the nation's children. Journal for Nurse Practitioners. 2007;3(10):713-720. doi: 10.1016/j.nurpra.2007.08.025.
137. Gans-Epner J, Brey L. Improving the delivery of clinical preventive services in school-based health centers (SBHCs). Journal of Adolescent Health. 1997;20(2):159.
138. Lecavalier L, Smith T, Johnson C, et al. Moderators of parent training for disruptive behaviors in young children with Autism Spectrum Disorder. Journal of Abnormal Child Psychology. 2017;45(6):1235-1245.doi: 10.1007/s10802-016-0233-x.
139. Bearss K, Johnson C, Smith T, et al. Effect of parent training vs parent education on behavioral problems in children with autism spectrum disorder: A randomized clinical trial. JAMA. 2015;313(15):1524-1533. doi: 10.1001/jama.2015.3150 .
140. Kulkarni S, Hall I, Formica R, et al. Transition probabilities between changing sensitization levels,waitlist activity status and competing-risk kidney transplant outcomes using multi-state modeling. PLoS One. 2017;12(12):e0190277. doi: 10.1371/journal.pone.0190277.
141. Sawyer B. In: Koster R, ed. A theory of fun for game design. Scottsdale, AZ: Paraglyph Press; 2005.
142. Sawyer B. From cells to cell processors: The integration of health and video games. Computer Graphics and Applications, IEEE. 2008;28(6):83-85. doi: 10.1109/MCG.2008.114.
143. Schell J. The Art of Game Design: A Book of Lenses. Burlington, MA: Morgan Kaufmann; 2008.
144. McCollister KE, Yang X, Sayed B, French MT, Leff JA, Schackman BR. Monetary conversion factors for economic evaluations of substance use disorders. Journal of Substance Abuse Treatment. 2017;81:25-34. doi: 10.1016/j.jsat.2017.07.008.
145. Murphy SM, Leff JA, Linas BP, Morgan JR, McCollister K, Schackman BR. Implementation of a nationwide health economic consultation service to assist substance use researchers: Lessons learned. Substance Abuse. 2018:1-19. doi: 10.1080/08897077.2018.1449173.
146. Murphy SM, McCollister KE, Leff JA, et al. Cost-effectiveness of buprenorphine-naloxone versus extended-release naltrexone to prevent opioid relapse. Annals of Internal Medicine. 2019;170(2):90-98. doi: 10.7326/M18-0227.
147. Kann L, McManus T, Harris WA, et al. Youth Risk Behavior Surveillance -- United States, 2017. Centers for Disease Control and Prevention; June 15, 2018 2018. doi: 10.15585/mmwr.ss6708a1.
148. Sorosiak D, Metcalf Walsh B, Peng J. Connecticut Youth Tobacco Survey Results; 2017 Surveillance Report. Hartford, CT 2018.
149. Kroenke K, Spitzer RL, Williams JB. The Patient Health Questionnaire-2: Validity of a two-item depression screener. Medical Care. 2003;41(11):1284-1292. doi: 10.1097/01.MLR.0000093487.78664.3C.
150. Spitzer RL, Kroenke K, Williams J, Lowe B. A brief measure for assessing generalized anxiety. Archives of Internal Medicine. 2006;166:1092-1097. doi: 10.1001/archinte.166.10.1092.
151. Curry LA, Nembhard IM, Bradley EH. Qualitative and mixed methods provide unique contributions to outcomes research. Circulation. 2009;119(10):1442-1452. doi: 10.1161/circulationaha.107.742775.
152. Morgan DL. Focus groups. Annual Review of Sociology. 1996;22:129-152.

153. Hergenrather KC, Rhodes SD, Cowan CA, Bardhoshi G, Pula S. Photovoice as community-based participatory research: A qualitative review. American Journal of Health Behavior. 2009;33(6):686-698.
154. Sibeoni J, Costa-Drolon E, Poulmarc'h L, et al. Photo-elicitation with adolescents in qualitative research: An example of its use in exploring family interactions in adolescent psychiatry. Child & Adolescent Psychiatry & Mental Health. 2017;11(49). doi: 10.1186/s13034-017-0186-z.
155. Glaser B, Strauss A, eds. The discovery of grounded theory: Strategies for qualitative research. Chicago: Aldine; 1967.
156. Crabtree B, Miller W, eds. Doing qualitative research. 2nd ed. Newbury Park, CA: Sage; 1999.
157. Hsieh HF, Shannon SE. Three approaches to qualitative content analysis. Qualitative Health Research. 2005;15(9):1277-1288. doi: 10.1177/1049732305276687.
158. Moullin JC, Dickson KS, Stadnick NA, Rabin B, Aarons GA. Systematic review of the Exploration, Preparation, Implementation, Sustainment (EPIS) framework. Implementation science: IS. 2019;14(1):1. doi: 10.1186/s13012-018-0842-6.
159. McGovern MP, Saunders EC, Kim E. Substance abuse treatment implementation research. Substance Abuse Treatment 2013;44(1):1-3. doi: 10.1016/j.jsat.2012.09.006.
160. Garner BR. Research on the diffusion of evidence-based treatments within substance abuse treatment: A systematic review. Journal of Substance Abuse Treatment. 2009;36(4):376-389. doi: 10.1016/j.jsat.2008.08.004.
161. Aarons GA, Hurlburt M, Horwitz SM. Advancing a conceptual model of evidence-based practice implementation in public service sectors. Administration and Policy in Mental Health. 2011;38(1):4-23. doi: 10.1007/s10488-010-0327-7.
162. U.S. Department of Health and Human Services Office of Adolescent Health. A Day in the Life. 2018; https://www.hhs.gov/ash/oah/facts-and-stats/day-in-the-life/index.html. Accessed January 25, 2019.
163. Substance Abuse and Mental Health Services Administration Center for the Application of Prevention Technologies. CAPT Decision-Support Tools: Preventing Prescription Drug Misuse: Understanding Who Is at Risk. 2016; https://www.samhsa.gov/capt/sites/default/files/resources/preventingprescription-drug-misuse-understanding.pdf. Accessed March 3, 2019.
164. Faggiano F, Minozzi S, Versino E, Buscemi D. Universal school-based prevention for illicit drug use. Cochrane Database of Systematic Reviews. 2014;12(CD003020):DOI: 10.1002/14651858.CD14003020.pub14651853.
165. Lennox RD, Cecchini MA. The NARCONON™ drug education curriculum for high school students: A non-randomized, controlled prevention trial. Substance Abuse Treatment, Prevention, and Policy. 2008;3(8):doi:10.1186/1747-1597X-1183-1188.
166. Kernan WN, Viscoli CM, Makuch RW, Brass LM, Horwitz RI. Stratified randomization for clinical trials.
Journal of Clinical Epidemiology. 1999;52(12):1289-1290.
167. McCabe SE, Morales M, Cranford JA, Delva J, McPherson MD, Boyd CJ. Race/ethnicity and gender differences in drug use and abuse among college students. Journal of Ethnicity in Substance Abuse. 2007;6(2):75-95. doi: 10.1300/J233v06n02_06.
168. Bruckner TA, Domina T, Hwang JK, Gerlinger J, Carpenter C, Wakefield S. State-level education standards for substance use prevention programs in schools: A systematic content analysis. Journal of Adolescent Health. 2014;54(4):467-473. doi: 10.1016/j.jadohealth.2013.07.020.
169. Nunes EV, Ball S, Booth R, et al. Multisite effectiveness trials of treatments for substance abuse and co-occurring problems: Have we chosen the best designs? Journal of Substance Abuse Treatment. 2010;38(Suppl 1):S97-112. doi: 10.1016/j.jsat.2010.01.012.
170. Compton WM, Jones CM, Baldwin GT. Relationship between nonmedical prescription-opioid use and heroin use. New England Journal of Medicine. 2016;374(2):154-163. doi: 10.1056/NEJMra1508490.
171. Saunders RP, Evans MH, Joshi P. Developing a process-evaluation plan for assessing health promotion program implementation: a how-to guide. Health Promotion Practice. 2005;6(2):134-147. doi:

10.1177/1524839904273387.
172. Skenderian JJ, Siegel JT, Crano WD, Alvaro EE, Lac A. Expectancy change and adolescents' intentions to use marijuana. Psychology of Addictive Behaviors. 2008;22(4):563-569. doi: 10.1177/1049732305276687.
173. Schwinn TM, Schinke SP, Di Noia J. Preventing drug abuse among adolescent girls: outcome data from an internet-based intervention. Prevention Science. 2010;11(1):24-32. doi: 10.1007/s11121-009-0146-9.
174. Callas PW, Flynn BS, Worden JK. Potentially modifiable psychosocial factors associated with alcohol use during early adolescence. Addictive Behaviors. 2004;29:1503-1515. doi: 10.1016/j.addbeh.2004.02.028.
175. Goldstein AP, McGinnis E. Skillstreaming the adolescent: New strategies and perspectives for teaching prosocial skills. Champaign, Illinois: Research Press; 1997.
176. Brooke J. SUS-A quick and dirty usability scale. In: Jordan PW TB, Weerdmeester BA. Usability Evaluation in Industry. London: Taylor & Francis; 1996:189-194.
177. O'Brien HL, Cairns P, Hall M. A practical approach to measuring user engagement with the refined user engagement scale (UES) and new UES short form. International Journal of Human-Computer Studies. 2018;112:28-39. doi: 10.1016/j.ijhcs.2018.01.004.
178. Fairchild AJ, MacKinnon DP. A general model for testing mediation and moderation effects. Prevention Science. 2009;10(2):87-99. doi: 10.1007/s11121-008-0109-6.
179. MacKinnon DP, Fairchild AJ, Fritz MS. Mediation Analysis. Annual Review of Psychology. 2007;58:593.
180. Aroian LA. The probability function of the product of two normally distributed variables. Annals of Mathematical Statistics. 1944/1947;18:265-271. doi: 10.1214/aoms/1177730442.
181. Sobel ME. Asymptotic intervals for indirect effects in structural equations models. In: Leinhart S, ed. Sociological methodology 1982. San Francisco: Jossey-Bass; 1982. doi: 10.2307/270723.
182. MacKinnon DP, Lockwood CM, Hoffman JM, West SG, Sheets V. A comparison of methods to test Mediation and other intervening variable effects. Psychological Methods. 2002;7:83-104.
183. Kraemer H, Wilson G, Fairburn C, Agras W. Mediators and moderators of treatment effects in randomized clinical trials. Archives of General Psychiatry. 2002;59:877-883.
184. Neumann PJ, Sanders GD, Russell LB, Siegel JE, Ganiats TG. Cost-Effectiveness in Health and Medicine, 2nd Ed. New York, NY: Oxford University Press; 2017.
185. Bureau of Labor Statistics. National compensation survey 2018. 2018; https://www.bls.gov/ncs/. Accessed February 17, 2019.
186. McCabe SE, Veliz PT, Boyd CJ, Schepis TS, McCabe VV, Schulenberg JE. A prospective study of nonmedical use of prescription opioids during adolescence and subsequent substance use disorder symptoms in early midlife. Drug & Alcohol Dependence. 2019;194:377-385. doi: 10.1016/j.drugalcdep.2018.10.027.
187. McCabe SE, West BT, Boyd CJ. Medical use, medical misuse, and nonmedical use of prescription opioids: Results from a longitudinal study. Pain. 2013;154(5):708-713. doi: 10.1016/j.pain.2013.01.011.
188. The White House Council of Economic Advisers. The Underestimated Cost of the Opioid Crisis. 2017;https://www.whitehouse.gov/briefings-statements/cea-report-underestimated-cost-opioid-crisis/. Accessed February 17, 2019.

189. Drummond MF, O'Brien B, Stoddart GL, Torrance GW. Methods for the Economic Evaluation of Health Care Programs. Second ed: Oxford University Press; 1997.
